# Economic evaluation of a complex intervention (Engager) for prisoners with common mental health problems, near to and after release: a cost-utility and cost-consequences analysis

**DOI:** 10.1007/s10198-021-01360-7

**Published:** 2021-08-05

**Authors:** Rachael Maree Hunter, Rob Anderson, Tim Kirkpatrick, Charlotte Lennox, Fiona Warren, Rod S. Taylor, Jenny Shaw, Mark Haddad, Alex Stirzaker, Mike Maguire, Richard Byng

**Affiliations:** 1 Research Department of Primary Care and Population Health, University College London (UCL), Rowland Hill Street, London, NW3 2PF UK; 2 Primary Care Department, College of Medicine and Health, University of Exeter, Exeter, UK; 3 Division of Psychology and Mental Health, University of Manchester, Manchester, UK; 4 University of Glasgow, Glasgow, UK; 5 City, University of London, London, UK; 6 NHS England, South West Mental Health Clinical Network, Taunton, UK; 7 Centre for Criminology, University of South Wales, Pontypridd, UK; 8 Community and Primary Care Research Group, University of Plymouth, Plymouth, UK

**Keywords:** Cost-utility, Cost-consequences, Prison, Common mental health problems, Mentalisation therapy, I100

## Abstract

**Background:**

People in prison experience a range of physical and mental health problems. Evaluating the effectiveness and efficiency of prison-based interventions presents a number of methodological challenges. We present a case study of an economic evaluation of a prison-based intervention (“Engager”) to address common mental health problems.

**Methods:**

Two hundred and eighty people were recruited from prisons in England and randomised to Engager plus usual care or usual care. Participants were followed up for 12 months following release from prison. The primary analysis is the cost per quality-adjusted life year (QALY) gained of Engager compared to usual care from a National Health Service (NHS) perspective with QALYs calculated using the CORE 6 Dimension. A cost-consequences analysis evaluated cross-sectoral costs and a range of outcomes.

**Results:**

From an NHS perspective, Engager cost an additional £2737 per participant (95% of iterations between £1029 and £4718) with a mean QALY difference of − 0.014 (95% of iterations between − 0.045 and 0.017). For the cost-consequences, there was evidence of improved access to substance misuse services 12 months post-release (odds ratio 2.244, 95% confidence Interval 1.304–3.861).

**Conclusion:**

Engager provides a rare example of a cost-utility analysis conducted in prisons and the community using patient-completed measures. Although the results from this trial show no evidence that Engager is cost-effective, the results of the cost-consequences analysis suggest that follow-up beyond 12 months post-release using routine data may provide additional insights into the effectiveness of the intervention and the importance of including a wide range of costs and outcomes in prison-based economic evaluations.

**Trial registration:**

(ISRCTN11707331).

**Supplementary Information:**

The online version contains supplementary material available at 10.1007/s10198-021-01360-7.

## Introduction

As of July 2020, there were approximately 80,000 people in prison in England and Wales, of which 96% are men and 95% are over the age of 18 [1]. People in prison experience significant physical and mental health problems compared to their peers in the community. People in prison are around 10 times more likely to have a mental health problem than the general population, with over half of the men in prison experiencing common mental health problems, which include anxiety, depression, phobias, obsessive compulsive disorder (OCD) and post-traumatic stress disorder (PTSD). Physical health care needs are also greater in the prison population and prisoners have higher mortality rates than their non-incarcerated peers [2]. The aim of prison though is punishment through the removal of liberty, maintaining the safety of the community and rehabilitation prior to release into the community, aims that can sometimes conflict with addressing the mental and physical health of people in prison [3]. The cause of the health concerns for people in prison may also originate from a range of sources including housing, employment, finances and relationships which require the involvement of multiple agencies to address. Meeting the physical and mental health care needs of people in prison population presents the health care, social care, welfare, housing and criminal justice agencies with challenges, both in terms of the logistics of working together to coordinate care as well as the significant resources required to meet the needs of this population.

Commissioning physical and mental health care in English prisons has been the responsibility of the National Health Service (NHS) since 2006 [4]. Mental health care in prison is provided by in-reach teams, with a number of models of delivery, the aim being to achieve an equivalence between mental health care in prisons and in the community. As a result there is some evidence that the mental health care in prisons is improving [5]. This care though covers specialist mental health care focussing on serious mental illness. In England, the diagnosis and treatment for common mental health problems is the responsibility of General Practitioners (GPs) and the Improving Access for Psychological Therapies (IAPT) service. However, the latter is not routinely delivered in prison settings. There is evidence that those in contact with the criminal justice system, whether in prison or the community, do not have their common mental health needs met: Byng et al. [6] found that 59% of people in contact with criminal justice had a common mental health problem, although only 61% felt they received the medication and 32% the therapy they needed. People also experience problems with continuity of care when they move from prison into the community.

In the United Kingdom (UK), there has been concern in the past regarding the financial pressure associated with providing health care in prisons [3]. In response, there has been an increased interest in identifying interventions that make best use of limited resources. In the UK, Her Majesty’s (HM) Treasury’s Green Book sets out the methodology for the evaluation of government-funded programmes. It specifically recommends the use of cost–benefit analysis (CBA), the monetary valuation of all costs and consequences of an intervention compared to current practice, for the evaluation of programmes over cost-effectiveness analysis (CEA), the cost per outcome gained of one intervention compared to another, due to the more restrictive nature of CEA [7]. Health care though is a noted exception given the difficulty of assigning monetary values to health outcomes. Instead, the recommended methodology for economic evaluations of health care interventions is a cost-utility analysis (CUA) calculated as the incremental cost per quality-adjusted life year (QALY) gained [7–10]. QALYs are calculated by combining information about mortality and morbidity into a single unit. This is to be done in a standardised way so as to allow for the comparison of costs and consequences across programmes of work and disease areas in health care, where best practice in England is to calculate morbidity from a preference-based tariff of health-related quality of life, most commonly using the Euroqol EQ-5D [8–10]. A systematic review conducted in 2017 of economic evaluations in the diagnosis and management of mental health problems for adults who are in contact with the criminal justice system did not identify any economic evaluations of prison interventions for common mental health problems or for mental health treatment more widely. Most economic evaluations instead focussed on programmes that divert people with serious mental illness away from prison or substance misuse treatment. The most common economic evaluation type was CEA, with no CUAs conducted [11]. Since the review was completed, the Critical time Intervention for Severely mentally ill prisoners (CrISP) study has been published which reported an array of cost information. Although the study collected resource use, there was no self-reported measure of health or quality of life included, hence a CUA was not conducted [12].

The aim of this paper is to report the results of an economic evaluation of the Engager intervention plus usual care compared to usual care using participant level trial data over 12 months following release from prison. Trial participants completed a range of patient-reported preference-based measures of mental and physical health-related quality of life and capability. They also completed a comprehensive battery of resource use questionnaires. The primary aim of the evaluation is to calculate the mean incremental cost per quality-adjusted life year (QALY) gained following release from prison and from an NHS perspective. The paper also investigates the cost impact of Engager plus usual care compared to usual care on a range of different public sector budgetary perspectives as well as including productivity gains. Costs are included alongside wider consequences to inform a cost-consequences analysis.

## Methods

Two investigation centres (south–west and north–west of England) recruited patients to a parallel, two-group, randomised control trial. Participants were randomised with an 1:1 allocation to either the Engager Intervention plus usual care (the intervention group) or usual care alone (the control group). Participants were included in the study if they were serving a prison sentence of 2 years or less in a male prison in England, with between 4 and 20 weeks remaining of their sentence and were identified as having or likely to have common mental health problems, including anxiety, depression, phobias, OCD and PTSD. Men were excluded if they met any of the following criteria: they were unable to provide consent; were on remand; had a serious and enduring mental health disorder including being on the caseload of the prison in-reach team; had a primary personality disorder; presented a serious risk of harm to the trial and intervention delivery team; or posed a risk of harm to themselves and the healthcare team felt participation in the study would be detrimental.

The primary objective of the trial was to evaluate the effectiveness of the Engager intervention in improving psychological and social outcomes for men with common mental health problems in prison, with a primary outcome of the Clinical Outcomes in Routine Evaluation Outcome Measure (CORE-OM) measured at 6 months after release from prison. The study was approved by the UK National Health Service, Wales Research Ethics Committee 3 (ref: 15/WA/0314) and the National Research Committee of Her Majesty’s Prison and Probation Service (ref 2015–283). Further details on the trial are available in the trial protocol [13], feasibility [14, 15] and results papers [16]. A number of trial processes and outcomes were informed by a feasibility trial on Engager run prior to the full trial.

### Engager intervention

Participants randomised to the intervention arm received the Engager intervention delivered by an Engager practitioner: a manualised, person centred intervention with the aim of meeting participants mental health needs. These included addressing wider support issues such as education, accommodation, social relationships and financial management that may be related to mental health. Prior to release the Engager practitioner worked with participants on goals and needs using a goal attainment plan. On release from prison ongoing work between the participant practitioner included signposting to key community services to address the participants needs. All of this was underpinned by practitioners offering a mentalisation-based approach to support.

Participants allocated to usual care continued with existing service provision for men prior to and following release from prison which included primary care, secondary care (specialist) mental health services, substance misuse services and other criminal justice and third-sector organisations that would provide support regarding education, accommodation, social relationships and financial management as standard.

### Cost of engager intervention

The cost of the Engager intervention includes the time of an Engager practitioner from a range of different disciplines including psychology, mental health nursing, substance misuse and housing at the level of assistant practitioner or entry level counsellor (NHS pay grade Band 4, £32 per hour [17]) to deliver the intervention, plus an allocation of the initial cost of training and supervision from a senior practitioner from a similar wide range of disciplines at Clinical Psychologist or Specialist level (NHS pay grade Band 7, £56 per hour [17]). Training and supervision costs were calculated as the time allocated to attend training sessions multiplied by the cost of practitioner and supervisor time; the cost of delivering the training and mentalisation-based approach (MBA) sessions; regular practitioner supervision; and meta-supervision conducted by a senior clinician (senior clinical consultant, £111 per hour [17]). As a conservative estimate (overestimate of the true cost if this was implemented as part of routine care at a larger scale), the cost per participant of training and supervision is calculated as the total cost of training and supervision for the whole Engager trial divided by the number of participants randomised to the intervention arm of the trial. Practitioners were directed to keep detailed records of the amount of time they spent delivering different aspects of the Engager intervention. This information was then transcribed into a database so that the cost of the intervention could be calculated for each participant in the intervention arm.

A top–down costing of the intervention has been included as a sensitivity analysis, costing staff involved in the intervention based on total full time equivalent staff including oncosts and overheads [17]. The total cost per participant is calculated as the total top–down cost of staff plus the additional cost of training divided by the number of participants in the intervention arm of the trial.

Data on additional services that participants were signposted to and attended as part of the intervention were collected using the resource use questionnaires, as described in the next section.

### Resource use and costs

Resource use in both groups was collected using a version of the Client Service Receipt Inventory (CSRI) [18] adapted based on the experience of the Engager feasibility trial [14]. The CSRI was broken into key areas with examples of services in each area provided. It also asked if the service use was planned or unplanned/emergency. Mental and physical health care (planned and emergency) including medication was self-reported at baseline, 6 months and 12 months post-release asking about the previous 3 months at baseline and since last follow-up at 6 and 12 months. Accommodation, education, training, employment, financial advice, relationship and criminal-related service use was self-reported at baseline, pre-release, 6 and 12 months post-release, asking about the previous 3 months at baseline and since last follow-up at pre-release, 6 and 12 months. Participants were asked to report number of contacts as well as average duration of contacts. Unit costs and sources used to calculate costs are reported in Table 1. Medication was costed using the British National Formulary [19].

**Table 1:** Resource use unit costs in 2017/2018 British Pounds

Resource use	Unit cost	References
Health care resource use		
Hospital transfer (community)	258	NHS Reference Costs [20]
Hospital transfer (prison)	4548	Department of Health [21]
Alcohol brief intervention (delivered by nurse)	8	PSSRU [17]
Community mental health nurse (per h)	34	PSSRU [17]
Counselling (per contact)	74	NHS Reference costs [20]
Criminal justice liaison service (per contact)	234	NHS Reference costs [20]
Dentist	164	NHS Reference costs [20]
Cognitive behavioural therapy (per contact)	74	NHS Reference costs [20]
GP (prison and community; per contact)	28	PSSRU [17]
Home help/care worker (per h)	28	PSSRU [17]
Substance misuse services: prison (per contact)	80	PSSRU prison [22]
Substance misuse services: community (per contact)	130	NHS Reference costs [20]
Mental health clinic (per contact)	160	NHS Reference costs [20]
NHS walk-in centres	35	Estimated using PSSRU [17]
Occupational therapist (per contact)	81	NHS Reference costs [20]
Optician (per contact)	54	Violato [23]
Peer groups for substance misuse (with Band 5 counsellor leading—per contact)	34	PSSRU [17]
Physiotherapist (per contact)	57	NHS Reference costs [20]
Practice nurse (per h)	37	PSSRU [17]
Prison nurse (per h)	37	PSSRU [17]
Psychiatrist (per h)	111	PSSRU [17]
Psychologist (per contact)	74	NHS Reference costs [20]
Sexual health worker (per contact)	120	NHS Reference costs [20]
Social worker (per h)	45	PSSRU [17]
Learning difficulties nurse (per contact)	79	NHS Reference costs [20]
Blood-borne viruses nurse	89	NHS Reference costs [20]
Behaviour change (per contact)	74	NHS Reference costs [20]
Pharmacy—dispensing cost (per contact)	9	PSNC [24]
Podiatrist/chiropodist (per contact)	51	NHS Reference costs [20]
Healthy living (per client)	120	PSSRU [17]
Smoking cessation (per contact)	15	NICE [25]
IAPT (per contact)	96	PSSRU [17]
Criminal justice		
Probation worker/community rehabilitation company (CRC) worker (per h)	21	Indeed Salaries [26]
Enhanced thinking skills	154	PSSRU Prison [22]
Healthy Relationships Programme (HRP) high (per contact)	148	PSSRU Prison [22]
HRP moderate (per contact)	121	PSSRU Prison [22]
Controlling anger (per contact)	114	PSSRU Prison [22]
Counselling, Assessment, Referral, Advice and Throughcare (CARAT) prison (per contact)	80	PSSRU Prison [22]
Education course (per attendance)	120	Ipsos Mori [27]
Prison (per person per year)	40,843	HM PPS [28]
Police (per contact)	457	Heslin [29]
Police (per night in custody)	411	Heslin [29]
Police (per additional day in custody)	1032	Heslin [29]
Local authority		
Citizens advice (per contact)	21^a^	Citizens Advice [30]
Employment worker/officer (per contact)	68	PSSRU 2015 [31]
Housing worker/officer (per contact)	25	Schneider [32]
Supported accommodation (per person per day)	118	PSSRU [17]
24 h supported accommodation (per person per day)	267	PSSRU [17]
Social housing (per person per week)	108	PSSRU [17]
Probation hostel (same as supported accommodation)	118	PSSRU [17]
Other		
Lawyer (per h)	200	Harcourt Barristers Direct [33]
Legal advocate (per contact)	34	Devine [34]
Listeners/visitors/samaritans (per contact)	49^b^	Samaritans [35]
Support from religious organisations (per h)	29^c^	Thornhill Parish Church [36]
Life coach (per contact)	50	Bidvine [37[

^a^£26.8 million in funding and 1,273,000 contacts

^b^£6.2 million in funding and £78 million in volunteer equivalent time; 3.6 million calls

^c^£230 per day to keep a church open

Traditionally employment costs are costed as wages or salary lost due to illness or interrupted employment. People in contact with criminal justice, however, have relatively low employment rates: prior to incarceration 33% of the Engager trial population were in paid employment. Rather than preventing the reduction of productivity through illness, the Engager intervention aims to facilitate access to paid employment. As a result, employment costs were costed as productivity gains using the human capital approach and assuming an hourly gross wage of £18.50 [38], which is the mean wage for men. Insufficient information was provided to use a job specific wage for each trial participant, but this value is close to the mean hourly wage for the construction industry (£17.29) [39], the most common area that the trial participants worked in where information was available. The total productivity gain per participant was then subtracted from total per participant costs.

All costs are reported in 2017/2018 British Pounds, the most recent year costing data were available for. Any costs for earlier years were adjusted for the current year using the hospital and community health services (HCHS) index for health and social care costs [17], and using the Services Producer Prices Index [39] for other costs.

### Outcome measures

Limited work has been done on determining suitable outcome measures for economic evaluations of interventions delivered in prisons. The choice of outcome measures for use in the trial was based on a Delphi exercise evaluating which outcome measures to use [40]. As part of the feasibility trial we identified which outcomes out of the CORE-OM [41], Euroqol EQ-5D 5 level (EQ-5D-5L) [42] and ICEpop CAPability Adult version (ICECAP-A) [43] were most sensitive to changes in the clinical measure of depression (PHQ-9) [44] and anxiety (GAD-7) [45], with CORE-OM being the most sensitive [15]. The CORE-OM was also chosen as the primary outcome of the main effectiveness analysis of the trial. The CORE-OM, a 34 item measure covering well being, problems/symptoms, life functioning and risk to self and others, designed to measure individual differences at the start of therapy and how these change over time [41], has an associated preference-based tariff, the CORE 6 Dimension (CORE-6D), that can be used in economic evaluations to calculate quality-adjusted life years (QALYs) [46]. As a result, our predetermined primary analysis for the economic evaluation was to calculate QALYs using the CORE-6D. The CORE-OM was collected at baseline, 1, 3, 6 and 12 months post-release from prison, applying the algorithm from Mavranezouli et al. [46] to calculate utility for the cost per QALY analysis. The EQ-5D-5L and ICECAP-A were collected at baseline, 3, 6 and 12 months post-release from prison. Utility from the EQ-5D-5L was calculated from (a) the van Hout mapping algorithm to the EQ-5D-3L recommended by that National Institute of Health and Care Excellence (NICE) [47]; (b) the EQ-5D-5L value set [48]. ICECAP-A capability was calculated based on the tariff developed by Flynn et al. [49].

### Statistical analysis

Analyses were pre-specified in a health economics analysis plan (HEAP see Supplementary Material 1).

We calculated complete case (participants that were followed up at that time point and completed that section of the questionnaire) descriptive statistics for the percentage of participants and mean number of contacts for each type of resource use. As questionnaires were completed with the aid of a research assistant, we assumed that if a value was missing for a resource use item it was because the participant did not use that item and hence it was imputed as 0. Questionnaires for participants that were followed that were specified as missing though were included as missing. Complete case means and standard deviations for costs were also calculated. The mean difference in costs, 95% confidence interval and *p* value for each resource use type was calculated using regression analysis adjusting for baseline costs, with centre as a covariate and bias-corrected bootstrapping with 3000 iterations for complete cases (available at all time-points).

QALYs were calculated as the area under the curve [10] using the CORE-6D and EQ-5D-5L. People that died before they reached a specific follow-up point are included as 0 for each follow-up point after they died, assuming a straight line from their last complete questionnaire until death. Years of Full Capability (YFC) (equivalent) were calculated using the ICECAP-A and methods for decision-making set out by the University of Birmingham [50]. For the CORE-6D, EQ-5D-5L and ICECAP-A, we report the mean values at each time point and mean unadjusted QALYs/YFC from baseline to 12 months. Mean difference in QALYs and YFC, 95% confidence interval and *p* value were calculated using regression analysis adjusting for baseline utility/tariff [10], with centre as a covariate and bias-corrected bootstrapping with 3000 iterations for complete cases (available at all time-points).

We assumed that data missing at follow-up was missing at random. Following examination of a range of outcome measures, we were unable to identify any predictors of missingness. Costs, utility scores and the ICECAP-A tariff were imputed for the recommended number of 30 datasets using chained equations (multiple imputation using chained equations (MICE)) and predictive mean matching [51].

For the incremental cost-effectiveness ratio (ICER), we use seemingly unrelated regression (Stata command SUREG) to account for the correlation between costs and outcomes to calculate the incremental mean cost per QALYs/YFC gained of Engager plus usual care compared to usual care. We adjusted for baseline and with centre as a covariate. The primary analysis was calculated using the multiple imputation dataset and bootstrapped results as set out by Leurant et al. [52]. The bootstrapped, imputed results were used to calculate the CEAC [53, 54]: the probability that Engager is cost-effective compared to usual care for a range of thresholds for a QALY/YFC gained. A cost-effectiveness plane has also been reported.

As the trial-based analysis covers a 12-month duration, no discount rate was applied. Analyses were conducted using Stata version 16 [55].

### Secondary within-trial analyses

ICERs, CEACs and CEPs will be reported for the following analyses:

(i) Health and social care cost perspective using the EQ-5D-5L for the calculation of QALYS.
(ii) Health and social care cost perspective using the ICECAP-A for the calculation of YFC.
(iii) All costs minus productivity gains and the CORE-6D for the calculation of QALYS
(iv) All costs minus productivity gains and the EQ-5D-5L for the calculation of QALYS
(v) All costs minus productivity gains and the ICECAP-A for the calculation of YFC.

### Sensitivity analyses

1. The Engager intervention is costed based on information on contact times reported by Engager practitioners. In sensitivity analysis 1, the Engager intervention is costed as the top–down costing that includes the total cost of employing practitioners based on their FTE. This reflects the actual cost to the NHS of delivering Engager, including the learning curve of delivering the Engager intervention, as well as tasks that may not have been reported by the practitioner, particularly administrative tasks.
2. If Engager were to be rolled out more widely, the meta-supervision delivered by a senior clinician is unlikely to be included as part of the training and supervision included in the cost of the intervention. As a result, we conducted a sensitivity analysis where the cost of meta-supervision was not included in the training and supervision cost.
3. In the Engager manual, it was stated that supervision was to occur on a weekly basis; in reality, it may occur less frequently than this, for example on a fortnightly basis. We include a sensitivity analysis with fortnightly supervision instead of weekly in the training and supervision costs.
4. Removing pre-release costs from the total costing as potentially these occurred before participants had received the Engager intervention.
5. There may be an interaction between being randomised to the Engager intervention and the pre-release duration in prison and other outcomes. A sensitivity analysis will include adjusting for the duration in prison pre-release, included as a covariate in the regression analysis.

### Cost-consequences analysis

Cost-consequences analysis facilitates the comparison between costs and a range of outcomes. This is particularly important for interventions such as Engager where different costs and consequences are likely to fall on a number of different public sector budget holders including health care, criminal justice and local government, who in England are responsible for substance misuse, social care and some accommodation services. Modelling work carried out prior to the trial also identified the importance of differentiating between planned versus unplanned care as a determinant of future costs and outcomes [56].

Our initial aim prior to obtaining trial data was to estimate the incremental cost of health and social care including the cost of the Engager intervention in the treatment arm compared with the incremental number of trial participants who had outcomes such as stable accommodation, were in employment or had reduced contact with criminal justice agencies. The cost and QALY benefits associated with these positive gains would then be extrapolated further into the future. Within the pre-specified HEAP, this analysis was given very broad methodological details as many aspects were reliant on the final results.

The analysis carried out as part of the main trial evaluation showed no evidence for participants randomised to the Engager intervention being more likely to be in stable accommodation [17]. There was also no evidence for reduced contact with criminal justice agencies, with the results suggesting instead the opposite. Instead, we chose to investigate if Engager compared to usual care resulted in any of the following:

- Greater odds of being in paid employment: calculated as an odds ratio adjusting for baseline employment and centre.
- Greater odds of accessing education: calculated as an odds ratio adjusting for baseline education and centre.
- Greater odds of accessing services to help with finance and accommodation: calculated as an odds ratio adjusting for baseline finance and accommodation service use, respectively.
- Greater odds of being in contact with substance misuse services: calculated as an odds ratio adjusting for baseline substance misuse need and centre.
- Reduced number of unplanned contacts: calculated using general linear models and family (Poisson or negative binomial) based on the most suitable model as informed by the Akaike Information Criterion (AIC) [57].

The weakness of this approach is that there is limited evidence on which to base any potential extrapolation of the benefits associated with each of these outcomes. The strongest evidence comes from improved access to substance misuse services. Although this may seem counterintuitive as it implies that the person has a substance misuse issue, for a population with a high level of substance misuse need this is an extremely important outcome: access to substance misuse services has been shown to be associated with reduced criminal activity and improved access to stable housing in the long term [58].

## Results

**Fig. 1:**
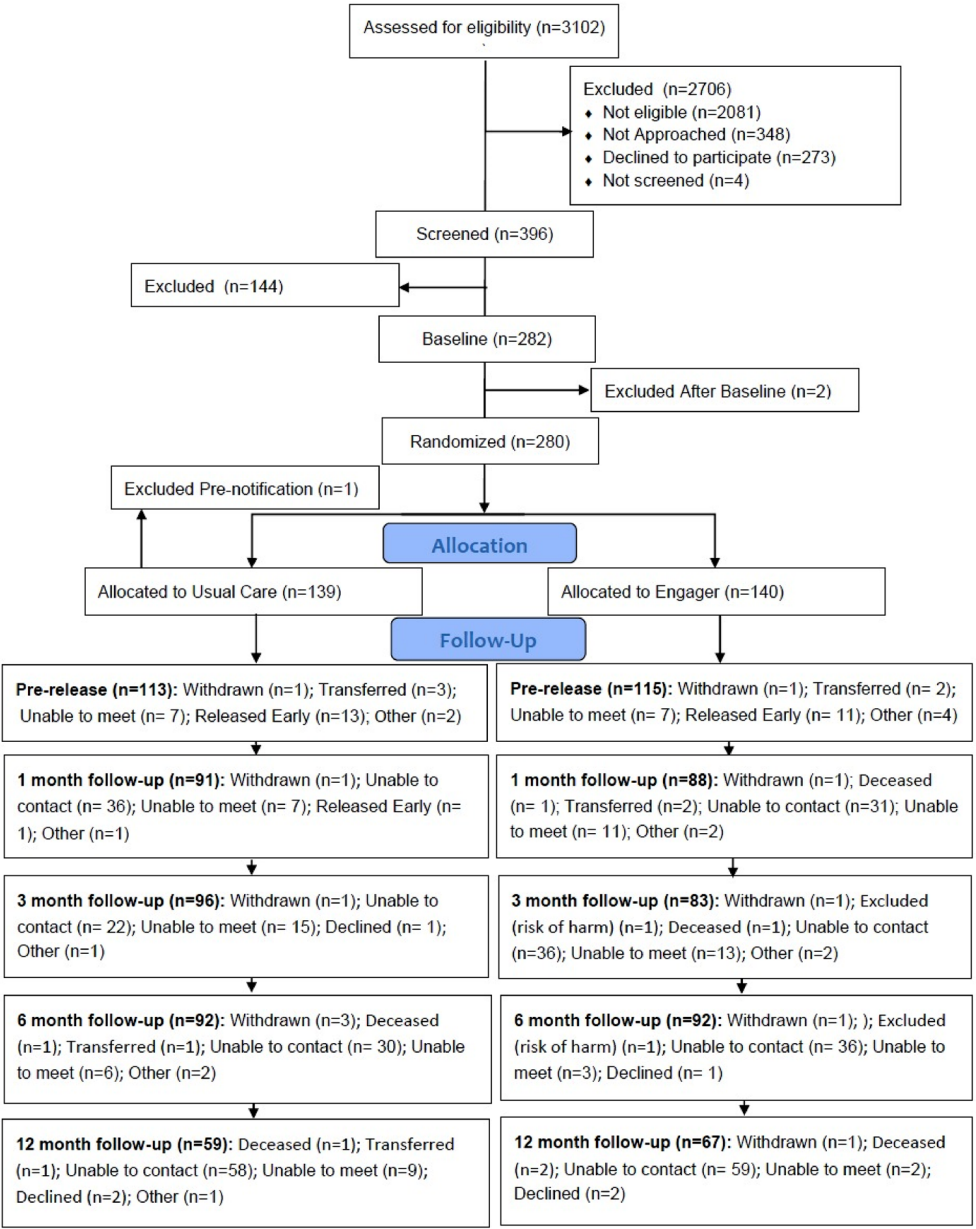
Engager consort diagram

Between January 2016 and October 2017, 280 eligible participants were identified and gave consent to be involved in the trial: 140 participants were randomised to Engager plus usual care and 140 to usual care (see Fig. 1 for Consort), with 1 person excluded post-randomisation in usual care (total 139 in usual care). Baseline characteristics of trial participants can be found in Table 2, with further details reported in the main effectiveness paper [17]. There was an imbalance between the two groups at baseline in the proportion in stable accommodation pre-release and in paid employment pre-release, with usual care participants more likely to be in stable accommodation and/or paid employment.

**Table 2:** Baseline characteristics

Characteristic	Engager	Usual care
	*N* = 140	*N* = 139
Age (years); mean (SD)	34 (11.4)	35 (9.9)
Ethnic group; *n* (%)		
White	128 (93)	133 (96)
Other	10 (8)	6 (4)
Pre-prison accommodation; *n* (%)		
Stable	56 (40)	73 (52)
Unstable	76 (54)	58 (41)
Enforced	8 (6)	8 (6)
Other	0 (0)	1 (1)
Educational background; *n* (%)		
No qualifications	38 (27)	34 (24)
Basic school level qualifications	41 (29)	41 (29)
A’ level or equivalent	10 (7)	12 (9)
Degree/professional qualification	51 (36)	53 (38)
Pre-prison employment status; *n* (%)		
Full-time/part-time paid employment	28 (20)	40 (29)
Full-time/part-time self employed	7 (6)	13 (9)
Other (e.g. voluntary, retired, carer)	1 (1)	2 (1)
Not working	104 (74)	85 (61)
Pre-prison income source; *n* (%)		
No source of income	22 (16)	11 (8)
Employment	30 (21)	40 (29)
Benefits	77 (55)	78 (56)
Other	11 (7)	11 (8)
Pre-prison income (£); *n* (%)	*N* = 138	*N* = 138
Less than 13,500	114 (82)	107 (76)
13,501 or more	24 (17)	31 (23)
Alcohol problem (self-report); *n* (%)	50/139 (36)	50 (36)
Drug problem (self-report); *n* (%)	69/139 (50)	60 (43)
CORE-6D; mean (SD)	0.750 (0.168)	0.713 (0.181)
EQ-5D-5L Crosswalk; mean (SD)	0.679 (0.234)	0.657 (0.225)
EQ-5D-5L Tariff; mean (SD)	0.767 (0.186)	0.754 (0.182)
ICECAP-A; mean (SD)	0.613 (0.221)	0.613 (0.226)

### Cost of the engager intervention

The total cost of training and supervision for the duration of the Engager trial was £59,303 (see Table 3). Of the 140 participants randomised to Engager, 129 are included in the intervention delivery cost analysis after removing withdrawals (*n* = 5), deaths during intervention delivery (*n* = 2) and participants where no case notes were available (*n* = 4). If the total cost of the training and supervision is divided by the 140 participants randomised to the intervention, the training and supervision costs £424 per participant. We have used the number of participants randomised to the intervention to calculate the cost per participant of training and supervision as this is more likely to reflect the total number of patients receiving the intervention if rolled out, if still a conservative estimate (over estimate of costs) as in reality this number would be higher.

**Table 3:** Total cost of training and supervision

Activity	Description	Total cost
Training attendance	3 training sessions delivered over 7 days attended by all 4 practitioners and 2 supervisors	£8400
MBA session attendance	8 MBA sessions attended by 4 practitioners and 2 supervisors	£5760
Training session for new staff	1 training session over 2 days for the 4 new practitioners and 1 new supervisor	£1840
Delivery of training	Cost of trainer to deliver all 9 sessions	£3375
Delivery of MBA session	Cost of trainer to deliver 8 MBA sessions	£2400
Weekly supervision	As directed by the Engager manual, weekly supervision over 2 years and accounting for 1 practitioner for 3 months in site 1 and no practitioners in site 2 for 3 months	£33,088
Meta-supervision	40 h of face-to-face and phone call contacts over the trial by a senior clinical academic	£4440
Total cost of training and supervision		£59,303

The cost per participant of the Engager intervention is detailed in Table 4. Of the 129 participants where data are available, on average they received 5.7 (SD 3.9) sessions in prison, with 5 participants (4%) having no contacts with practitioners. The average time per session delivered in prison was 43 min (SD 17) with an average cost of the prison component of the intervention of £149 (SD 124) per trial participant, including those who did not have any contact with a practitioner in prison. Including only participants that had at least one session with a practitioner in prison, the average cost per participant is £155 (SD 122). Of the 129 participants that we have data available for, 61 (47%) were met ‘at the gate’ (soon after release) by an Engager practitioner, and another 10 (8%) had another form of ‘at the gate’ contact (phone call or probation), with an average contact time of 215 min (SD 128) and an average cost of £61 (SD 76) per participant, including those who did not have any contact with an intervention practitioner ‘at the gate’. A total of 108 (84%) of participants received at least one Engager session in the community with the average session time for the interventions delivered in the community being 36 min (SD 42) per session (face-to-face and telephone contacts). The average cost per participant of the community component of the intervention was £256 (SD 384), including those who did not have any contact with a practitioner. The total average cost per participant of delivering all intervention sessions (prison, ‘at the gate’ and community) was £467 (SD 475). When the cost of training and supervision (£424) is added, this is an average cost per participant in the Engager arm of £891.

**Table 4:** Multiple imputation by chained equations (MICE) mean cost and adjusted difference

	Engager		Usual care					
	*N* = 129							
	mean	SD						
Cost of the intervention								
Training and supervision (per person)	424							
Prison component	149	124						
At release	61	76						
Community	256	384						
Total cost per participant (exc training)	467	475						
Total cost per participant (inc training)	891	475						

	*N* = 140		*N* = 139		Adjusted difference^a^	95% CI	95% CI	*p* value
	Mean	SE	Mean	SE				
Specialist mental health								
6 months	116	47	67	30				
12 months	965	592	21	10				
Total (unadjusted)	1081	598	88	32				
Total (adjusted)	1071	503	98	327	973.139	− 209.542	2155.820	0.106
Physical health inpatient-planned								
6 months	17	17	158	147				
12 months	284	116	0					
Total (unadjusted)	302	117	158	147				
Total (adjusted)	301	142	158	122	143.458	− 226.278	513.195	0.445
Physical health inpatient-unplanned								
6 months	300	86	202	89				
12 months	342	137	393	149				
Total (unadjusted)	642	171	596	176				
Total (adjusted)	615	178	622	167	− 6.976	− 494.176	480.224	0.977
Outpatient appointments								
6 months	50	22	38	17				
12 months	38	17	63	31				
Total (unadjusted)	88	27	101	33				
Total (adjusted)	87	30	102	31	− 15.231	− 100.520	70.059	0.724
Community health care								
Pre-release	371	71	316	63				
6 months	1105	161	913	177				
12 months	1483	285	1722	431				
Total (unadjusted)	2959	373	2951	502				
Total (adjusted)	2942	406	2969	466	− 26.637	− 1195.089	1141.815	0.964
Medication								
Total (unadjusted)	196	152	58	18				
Total (adjusted)	193	108	61	109	132.695	− 169.549	434.939	0.388
Total health care								
6 months	1838	232	1515	289				
12 months	3783	750	2257	476				
Total health care (unadjusted)	5019	812	3842	733				
Total (adjusted)	5937	787	4144	687	1793.183	− 257.042	3843.409	0.086
Total inc. Engager (adjusted)	6789	788	4146	688	2643.315	590.127	4696.502	0.012
Criminal justice service use								
Pre-release	30	23	35	31				
6 months	125	17	160	31				
12 months	135	25	272	118				
Total (unadjusted)	289	40	467	128				
Total (adjusted)	291	73	465	112	− 174.874	− 442.159	92.410	0.196
Prison								
6 months	5179	744	3712	709				
12 months	6141	1182	4021	861				
Total (unadjusted)	11,320	1568	7733	1108				
Total (adjusted)	11,314	1500	7739	1183	3574.627	− 104.371	7253.625	0.057
Police								
6 months	2130	460	1150	413				
12 months	2407	1249	1288	310				
Total (unadjusted)	4537	1332	2438	550				
Total (adjusted)	4499	1112	2476	912	2023.223	− 842.815	4889.261	0.165
Total CJS								
6 months	7434	906	5021	866				
12 months	8683	1709	5581	943				
Total (unadjusted)	16,146	2031	10,637	1305				
Total (adjusted)	16,057	1843	10,728	1540	5329.257	464.327	10,194.186	0.032
Total inc. Engager (adjusted)	16,894	1941	10,701	1534	6192.966	1083.378	11,302.550	0.018
Accommodation								
6 months	3153	809	2943	917				
12 months	4735	1594	5899	1718				
Total (unadjusted)	7888	1869	8842	1971				
Total (adjusted)	7886	1916	8844	1921	− 958.558	− 6428.064	4510.947	0.726
Productivity								
6 months	3560	1013	3095	808				
12 months	3921	1133	4870	1454				
Total (unadjusted)	7481	1509	7965	1837				
Total (adjusted)	8282	1514	7157	1816	1124.992	− 3491.543	5741.527	0.628
Education								
Pre-release	544	129	485	123				
6 months	117	45	49	29				
12 months	190	71	28	9				
Total (unadjusted)	852	149	563	128				
Total (adjusted)	849	138	566	138	282.865	− 92.505	658.236	0.139
Other services								
Pre-release	56	12	41	9				
6 months	470	106	229	45				
12 months	400	196	433	134				
Total (unadjusted)	926	239	704	147				
Total (adjusted)	926	214	703	182	222.838	− 318.854	764.531	0.416
All costs minus productivity								
Pre-release	1006	148	882	147				
6 months	9452	1738	6663	1722				
12 months	14,067	2765	9387	2245				
Total (unadjusted)	24,525	3376	16,932	3090				
Total (adjusted)	23,327	3254	18,138	3109	5189.067	− 3726.096	14,104.231	0.250
Total inc. Engager (adjusted)	24,177	3253	18,142	3108	6034.631	− 2878.161	14,947.420	0.182

*SD* standard deviation, *SE* standard error, *CI* confidence interval, *CJS* criminal justice system

^a^Adjusted difference: adjusted for baseline, with centre as a covariate

A second way to cost the intervention would be to use total staff wages and overheads. The delivery of the intervention required two whole full time equivalent (FTE) NHS Band 4 staff and one 0.5 FTE NHS Band 7 staff member at each site over the 2 years. Including oncosts and overheads as taken from PSSRU [18], the total cost for the two Band 4 Engager practitioners per site per year is £95,230 and the total cost of the Band 7 0.5 FTE supervisor per year is £45,370 for a total cost of £138,800 per site per year. Over two sites and two years, this is a total staff and overheads cost of £555,200. However, staff turn-over meant that the sites were not at their full staff profile for the whole 2 years: for 3 months, there was only one Band 4 staff member in one site and for a second 3 months, there were no Band 4 staff members at the other site, hence reducing Band 4 salaries by a 9-month period £35,711, the total revised cost is £519,488. The additional training costs on top of this (see Table 2: includes delivery of training and Meta-supervision) total £10,215, for a total cost of £529,704. Divided by 140 participants, this is a total cost per participant of £3784.

### Resource use and costs

Descriptive statistics for resource use are reported in Supplementary Material 2. Table 3 reports the mean costs, adjusted means and adjusted mean difference adjusting for baseline for each cost category and including centre as a covariate. The complete case results for costs are reported in Supplementary Material 3.

The mean difference in all health care costs including the cost of the Engager intervention and training is £2643 (95% CI £590–£4697) per participant, with missing data imputed using MICE and adjusting for baseline and centre. The adjusted imputed difference in criminal justice costs including the cost of the Engager intervention is £6193 (95% CI £1083–£11,303) per participant; for all costs minus productivity and including the costs of the intervention the imputed adjusted difference in costs is £6,035 (95% CI − £2,877 to £14,947) per participant.

### QALYs and capability gains

Descriptive statistics for the imputed CORE-6D, EQ-5D-5L (cross-walk and TTO tariff) and ICECAP-A tariff are reported in Table 5. There was no significant difference in QALYs or YFC for any of the analyses. The complete case results are reported in Supplementary Material 3.

**Table 5:** Multiple imputation by chained equations utilities, capability and QALYs,

	Engager		Usual care		Adjusted difference^a^	95% CI	95% CI	*p* value
	*N* = 140		C					
	Mean	SE	Mean	SE				
CORE-6D								
1 month	0.743	0.022	0.761	0.024				
3 months	0.741	0.020	0.768	0.020				
6 months	0.753	0.019	0.780	0.019				
12 months	0.752	0.024	0.693	0.036				
QALYs (unadjusted)	0.749	0.013	0.751	0.015				
QALYs (adjusted)	0.743	0.012	0.757	0.014	− 0.014	− 0.052	0.023	0.455
EQ-5D-5L Crosswalk								
3 months	0.695	0.026	0.679	0.028				
6 months	0.695	0.022	0.707	0.026				
12 months	0.733	0.031	0.631	0.042				
QALYs (unadjusted)	0.703	0.017	0.675	0.019				
QALYs (adjusted)	0.698	0.015	0.680	0.016	0.019	− 0.023	0.039	0.379
EQ-5D-5L Tariff								
3 months	0.778	0.021	0.765	0.022				
6 months	0.768	0.021	0.785	0.021				
12 months	0.796	0.028	0.757	0.034				
QALYs (unadjusted)	0.777	0.016	0.769	0.017				
QALYs (adjusted)	0.774	0.013	0.772	0.014	0.002	− 0.036	0.027	0.919
ICECAP-A								
3 months	0.636	0.026	0.632	0.027				
6 months	0.651	0.022	0.694	0.024				
12 months	0.690	0.037	0.707	0.031				
YFC (unadjusted)	0.652	0.016	0.673	0.018				
YFC (adjusted)	0.652	0.015	0.673	0.015	− 0.021	− 0.064	0.022	0.335

*SE* standard error, *CI* confidence interval, *QALYs* quality-adjusted life years, *YFC* years of full capability

^a^Adjusted difference: adjusted for baseline, with centre as a covariate

### ICER, CEAC and CEP

**Fig. 2:**
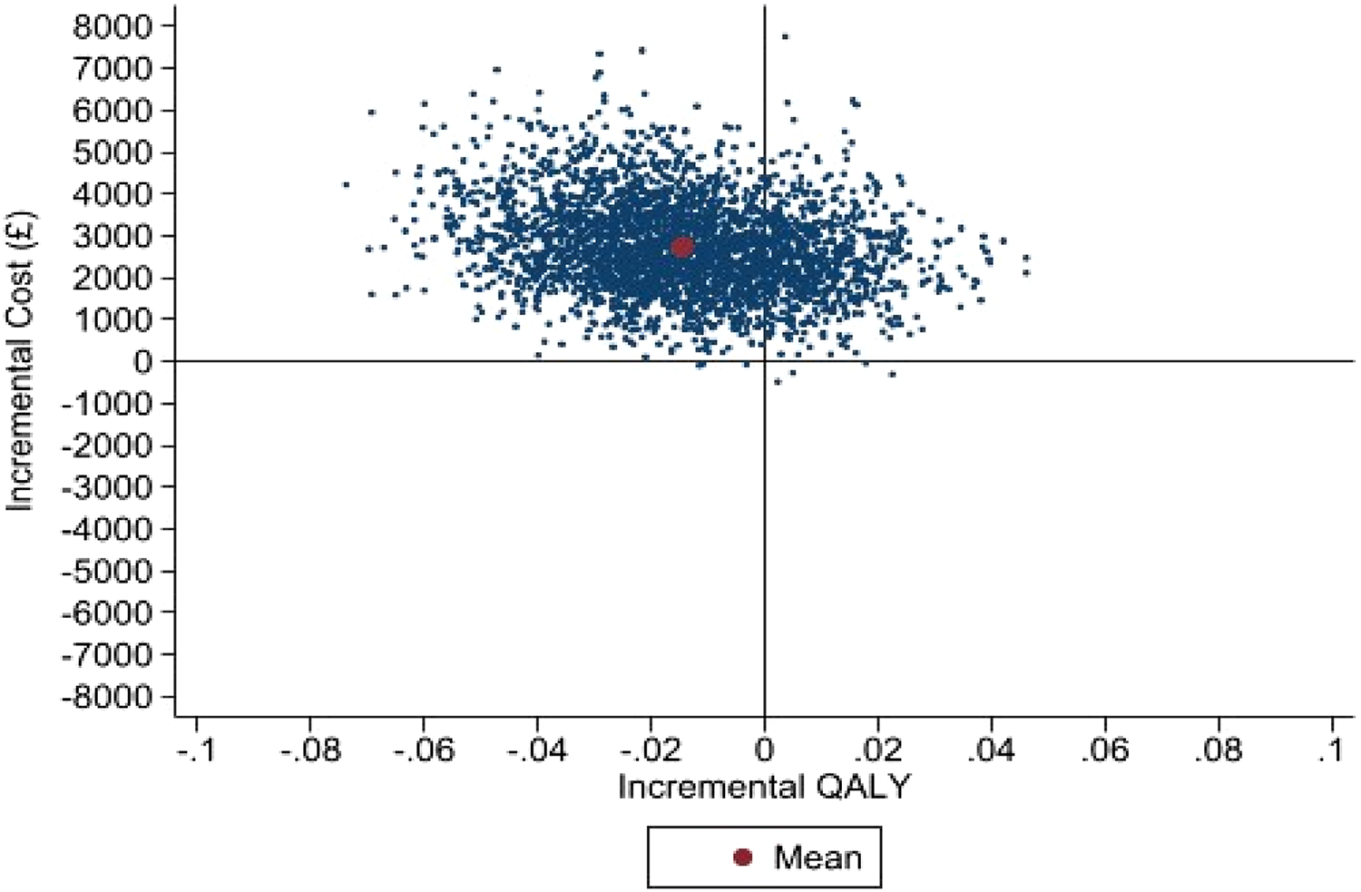
Cost-effectiveness plane of Engager compared to usual care from a health and social care cost perspective over 12 months with QALYs calculated using the CORE-6D

For the primary economic evaluation, within-trial cost-effectiveness analysis over 12 months, from a health and social care cost perspective with QALYs calculated using CORE-6D, MICE used for missing cost and utility data and seemingly unrelated regression to account for correlation between costs and outcomes, with adjustment for baseline and centre, there was a mean cost difference of £2738 (95% of iterations between £1030 and £4717) and a mean QALY difference of − 0.014 (95% of iterations between − 0.046 and 0.017): the Engager intervention is dominated by usual care. The CEP is reported in Fig. 2 and the CEAC in Supplementary Material 4. There is a 0% probability that the intervention is cost-effective for a £20,000 and £30,000 threshold for a QALY gained, if evaluated as a purely health care intervention where decision to implement would be decided along the standard conventions for other new technologies in the English NHS. The CEP and CEAC for the secondary and sensitivity analyses are reported in Supplementary Material 4. The conclusions remain consistent for all of the analyses conducted.

### Cost-consequences

Table 6 reports the results of the consequences component of the cost-consequences analysis. Paid employment and contact with service use are reported as odds; planned and unplanned service use were analysed using general linear models and either negative binomial or Poisson depending on the most appropriate model as indicated by the AIC [57].

**Table 6:** Consequences of Engager intervention compared to usual care: odds ratios and general linear models

	Engager		Usual care		OR	95% CI Lower	95% CI Upper	*p* value
	*n*	%(*n*)	*n*	%(*n*)				
Paid employment								
Pre-incarceration	140	25.71% (36)	140	39.29% (55)				
Release to 6 months	92	19.57% (18)	90	25.56% (23)	0.952	0.437	2.078	0.903
Release to 12 months	92	25.00% (23)	90	27.78% (25)	1.28	0.591	2.753	0.535
Contact with substance services								
Pre-baseline	140	33.57% (47)	140	34.29% (48)				
Baseline to pre-release	128	29.69% (38)	129	22.48% (29)	1.565	0.856	2.859	0.146
Baseline to 6 months post-release	136	44.12% (60)	135	31.11% (42)	2.085	1.197	3.633	0.010
Baseline to 12 months	136	49.26% (67)	135	34.07% (46)	2.244	1.304	3.861	0.004
Education								
Pre-baseline	140	43.57% (61)	140	35.71% (50)				
Pre-release	128	26.56% (34)	129	24.81% (32)	1.065	0.606	1.871	0.828
Baseline to 6 months	130	34.62% (45)	129	27.13% (35)	1.395	0.8188	2.376	0.221
Baseline to 12 months	130	36.15% (47)	129	27.13% (35)	1.484	0.873	2.523	0.145
Help with finances								
Pre-baseline	140	15.71% (22)	140	18.57% (26)				
Pre-release	128	23.44% (30)	129	19.38% (25)	1.337	0.708	2.524	0.370
Baseline to 6 months	136	43.38% (59)	135	40.00% (54)	1.143	0.704	1.855	0.589
Baseline to 12 months	136	47.79% (65)	135	47.41% (64)	1.010	0.626	1.629	0.967
Help with accommodation								
Pre-baseline	140	49.29% (69)	140	50.00% (70)				
Pre-release	128	25.78% (33)	129	22.48% (29)	1.336	0.680	2.625	0.400
Baseline to 6 months	136	46.32% (63)	135	42.22% (57)	1.223	0.729	2.053	0.445
Baseline to 12 months	136	53.68% (73)	135	48.15% (65)	1.277	0.776	2.101	0.336

Unplanned attendances	*n*	Mean (SD)	*n*	Mean (SD)	AD^b^	95% CI lower	95% CI upper	*p* value
Mental health								
Baseline	140	0.200 (0.614)	140	0.093 (0.414)				
6 months	92	0.196 (0.579)	90	0.100 (0.337)	0.487	− 0.407	1.381	0.285
12 months	66	0.182 (0.579)	58	0.0517 (0.223)	1.326	0.059	2.593	0.040
Physical health—unplanned								
Baseline	140	0.293 (0.594)	140	0.136 (0.344)				
6 months	92	0.446 (1.252)	90	0.211 (0.571)	0.723	0.089	1.358	0.025
12 months	66	0.197 (0.401)	58	0.397 (0.793)	− 0.701	− 1.381	− 0.020	0.043
Other services								
Baseline	140	1.814 (4.551)	140	2.179 (5.206)				
Pre-release	128	1.141 (3.578)	129	0.729 (1.291)	0.379	0.016	0.743	0.041
6 months	92	2.348 (6.591)	90	1.856 (7.602)	0.039	− 0.377	0.455	0.854
12 months	66	2.242 (12.449)	58	1.810 (4.847)	0.034	− 0.498	0.565	0.902

*OR* odds ratio adjusting for baseline and centre, *AD* adjusted difference, adjusting for baseline and centre, *CI* confidence interval

The odds of being in contact with substance misuse services were greater in the intervention group at 6 months after release (2.208 95% CI 1.197–3.633) and 12 months after release (2.244 95% CI 1.304–3.861). The results for unplanned service use are mixed, with more unplanned mental health contacts at 12 months after release in participants randomised to Engager (1.326 95% CI 0.059–2.593), an increase in physical health unplanned contacts at 6 months after release (0.723 95% CI 0.089–1.358), but a decrease in unplanned physical health contacts at 12 months after release (− 0.701 95% CI − 1.381 to − 0.020). Unplanned contact with other services was also higher in the Engager group pre-release (0.379 95% CI 0.016–0.743). There was no significant impact on the odds of being in paid employment, accessing education, access to help with finances or accommodation.

## Discussion

There was no evidence that the Engager intervention was cost-effective compared to usual care; this was the case across all secondary and sensitivity analyses. Overall, there was no significant difference in QALYs or YFC between Engager intervention arm participants and usual care participants. The intervention group cost significantly more from a health service cost perspective, with almost half of the estimated incremental cost per person coming from the Engager intervention itself. It also cost significantly more from other public service perspectives such as criminal justice. On the other hand, there was evidence for a productivity gain in the Engager group in the complete case analysis, although this difference was no longer significant in the imputed results and there was no evidence of increased employment in the analysis of consequences, suggesting this result may have been by chance.

Very few economic evaluations are carried out in criminal justice settings, with self-reported outcomes in this group being particularly hard to collect due to the transient nature of the population. This trial though presents a significant contribution to the health economic evidence base for this population group. It also demonstrates the complexity of economic evaluations in this area, with the results of the analysis having implications for a number of decision makers, including health care, criminal justice and local authorities. Costs and outcomes have been reported in a disaggregated way to facilitate interpretation by each respective decision maker, although no one decision maker is likely to advocate for implementing Engager based on these results. The complexity of providing services to multi-need clients across a number of public sector agencies is not new, with individuals with needs relating to mental health, physical health, housing, substance misuse, monetary and family relations being common in criminal justice, substance misuse and specialist mental health settings. Given that addressing one need, such as substance misuse, may have benefits that fall on other providers, such as criminal justice, initiatives such as pooled budgets across providers have been trialled to allow for the free flowing of money and outcomes across traditional barriers to facilitate joined up working [59]. Results from pilots of these interventions though have been equivocal in finding evidence for improved effectiveness or efficiency as a result of these initiatives [60, 61].

Although there was no evidence for benefit or cost-effectiveness for the Engager intervention, the cost-consequences analysis showed some signals for potential benefit, although the results are mixed. The most evident benefit was an increased odds of accessing substance misuse services seen in the participants randomised to Engager. Long-term studies such as the National Treatment Outcomes Research Study (NTORS) have shown the benefit of being in contact with substance misuse services in terms of reduced criminal activity and increased stable accommodation [58]. In NTORS, the evidence was that benefits accrued year on year over 5 years, hence the follow-up time of 12 months post-release in Engager may not have been sufficient to identify the benefits to participants. The loss to follow-up may have also failed to capture some benefits. In NTORS, routine data were used to capture criminal convictions. This was explored as part of Engager, but barriers to accessing data and time constraints in regards to the programme ending meant that this was not feasible. Ideally, routine data should be obtained for participants in the trial at a later time point to observe if there are any long-term benefits of the intervention.

As part of the HEAP, we made a predetermined choice for the primary CUA to calculate QALYs using the CORE-6D as opposed to using the NICE ‘reference case’ EQ-5D [9]. This was based on analyses undertaken during the feasibility trial that the CORE-OM and associated CORE-6D tariff are more sensitive to changes in the clinical measure of depression, the PHQ-9, in men in prison. Previous studies have found that the EQ-5D is acceptable for use in common mental health problems [62] and as the NICE ‘reference case’, it also allows for comparison of the results of economic evaluations across disease areas. There is an issue though when the EQ-5D is not sensitive to changes in a specific clinical condition and/or patient group that this may result in less favourable resource allocation decisions for those areas. There is some evidence that the EQ-5D may not be suitable in prison populations, but additional research is required to explore this further. The ICECAP-A, designed to measure wider considerations than health-related quality of life, also did not appear to capture anything additional in this population group. The results remain the same regardless though of the specific outcome measure used.

Overall, there is a challenge when evaluating interventions such as Engager which are designed to improve access to health and social care services for hard to reach groups. There is strong evidence that people who have spent time in prison are less likely to access health care services than their peers in the general population [6]. This group also strongly overlap with homeless and substance misuse populations who also show less health care service use relative to need than the wider population [63]. The consequences component of the cost-consequences analysis provided some evidence for increased access to services such as substance misuse services, but it also showed an increase in unplanned service use, particularly for specialist mental health services (noting that only one mental health service use was identified as planned). What is difficult to evaluate from this result is if this is a sign of improved access to mental health services as a result of the Engager intervention, or if the Engager intervention was linked to worsening mental health. In addition, to note that this is complicated by people in the Engager intervention group being a more severe group at baseline by chance, and although we adjusted for baseline mental health contacts this may have continued to skew the results.

In CUA, the aim is that additional costs arising from increased service use to meet identified needs is usually balanced out by additional gains in health-related quality of life, such that for new treatments NICE has a threshold of paying £30,000 per additional QALY gained [9]. There was no evidence though for improved QALYs or YFC as a result of the intervention. This may be due to the intervention not being effective in these areas, but it may also be due to these measures not being suitable in this population group, bias as a result of loss to follow-up or the time-horizon of the analysis being too short, particularly if in the short term the intervention required people to work through painful mental health or substance misuse problems.

One of the challenges associated with conducting research in prisons is loss to follow-up for patient-completed measures: this may explain why other trials in prisons have not included a preference-based outcome as part of their economic evaluation. The follow-up up rate of 66% for the primary clinical outcome at 6 months is high compared to most studies in prisons [17]. Contacting people following prison contains a range of issues including temporary housing, changing contact details as well as increased mental and physical problems that can make people difficult to contact. Unfortunately, there is also a nefarious aspect to this: those who may have returned to substance misuse and the criminal activities to support it may be more difficult to contact and actively want to avoid being contacted (although noting self-reported substance misuse problems at baseline were not a predictor of missing data at follow-up). Related to this, one of the more unexpected findings of the trial is that the Engager intervention group had significantly higher criminal justice costs. Measuring criminal activity is notoriously difficult; self-report measures of crime, in addition to being unreliable, may also have a negative impact on the relationship between the researcher and the trial participant, regardless of what reassurances of anonymity are provided [64]. Engager included no self-report measure of crime for this reason. The intention had been to obtain Police National Computer (PNC) data for the whole sample but this was in the end not possible. We could, therefore, only include the proxy measure of reported contact with police or being in custody. Although arguably more objective than self-report involvement in crime, this only measures if people are caught being involved in criminal activity, not the frequency with which the criminal activity occurs. One of the potential benefits of the Engager intervention is improved contacts with services; the implications of this may have been that this made people more visible to criminal justice agencies and hence more likely to be picked up for crimes. We were also unable to include implications of the wider costs, particularly to victims, of criminal activity.

Finally, there were some challenges in the delivery of the Engager intervention, with not all participants engaging with the intervention, and some discontinuity in the practitioners delivering the intervention due to staff turn-over and illness [16]. It is possible that embedding the training and delivery of Engager in already existing teams and making it part of normal delivery may reduce the cost of delivering the intervention and improve engagement. Further work is required to evaluate the implications for the cost and clinical effectiveness of these changes.

## Conclusion

The above economic evaluation is one of the few CUA conducted for a prison-based intervention. Although there was no evidence that the Engager intervention was cost-effective, it provides evidence for the feasibility of conducting CUA in this population. Future research though should consider supplementing the analysis with routine data and increasing the follow-up duration. It demonstrates the importance of including resource use and cost information to cover a wide array of decision makers as health interventions for prison have implications beyond health care, and reporting the results in a disaggregated way.

## Electronic supplementary material

Below is the link to the electronic supplementary material.

Supplementary material 1 (DOCX 86 kb)

Supplementary material 2 (PDF 438 kb)

Supplementary material 3 (DOCX 58 kb)

Supplementary material 4 (DOCX 442 kb)
