# Supplementary material for: Economic evaluation of a complex intervention (Engager) for prisoners with common mental health problems, near to and after release: a cost-utility and cost-consequences analysis
Source: Eur J Health Econ. 2021 Aug 5;23(2):193–210. doi: 10.1007/s10198-021-01360-7 (PMC8882099; doi:10.1007/s10198-021-01360-7)
Supplement: Supplementary file 1 — Supplementary material 1 (DOCX 86 kb) [file 10198_2021_1360_MOESM1_ESM.docx]

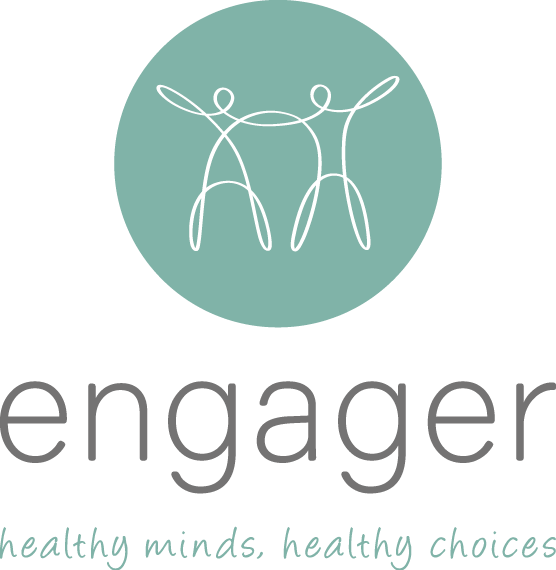


Evaluation of a complex intervention (Engager) for prisoners with common mental health problems, near to and after release – Full trial**.**

**Health Economic Analysis Plan (HEAP)**

**Version 0.1, 1^st^ February 2019**

Table of contents

[1. Aim 4](#_Toc291136)

[2. Outcomes 4](#_Toc291137)

[3. COST data 5](#_Toc291138)

[4. Quality of life data collection 7](#_Toc291139)

[5. Primary within-trial analysis 8](#_Toc291140)

[6. MISSING DATA 8](#_Toc291141)

[7. SECONDARY WITHIN-TRIAL ANALYSES 8](#_Toc291142)

[8. Cost Consequences ANALYSIS 9](#_Toc291143)

[9. Discounting 10](#_Toc291144)

[10. Sensitivity analysis 10](#_Toc291145)

[References 11](#_Toc291146)

# Aim

The primary aim of the health economic analysis is to calculate the mean incremental cost per quality adjusted life year (QALY) gained of the Engager intervention plus usual care compared to usual care. The primary analysis using patient level data will be for a 12 month time horizon and from a National Health Service (NHS) cost perspective. A secondary analysis from a public sector budgetary perspective (including: social services, police and criminal justice, national probation service, housing) will also be conducted to the extent that reliable and complete data from relevant services is available and usable.

A second aim is to conduct a cost-consequences analysis, reporting costs for the two groups alongside their respective outcomes.

The health economic analysis will follow the statistical analysis plan (SAP v1.4 December 2018) in retaining the validity of the randomisation process and Protocol version 5 (19/04/2017). All analyses will be intention-to-treat (ITT) where all randomised patients are analysed in their allocated group whether or not they received their allocated treatment. Although the primary outcome for the trial is at 6 months, the health economics analysis will only be undertaken using 12 month data.

# Outcomes

A full description of all outcomes and analysis are provided in the SAP and Trial Protocol.

The following outcomes will be used for the trial based component of the economic evaluation:

- Client services receipt inventory (CSRI)^1^ adapted – patient completed questionnaire asking about health care use (physical health and mental health) and medication completed at baseline, 6 and 12 months post release from prison asking about the last 6 months. The CSRI also includes details on the delivery of the intervention.
- CORE-OM baseline, 1, 3, 6 and 12 months post release from prison to calculate utility for cost per QALY analysis;
- EQ-5D-5L baseline, 3, 6 and 12 months post release from prison to calculate utility for cost per QALY analysis;
- ICECAP-A baseline, 3, 6 and 12 months post release from prison to calculate years of full capability equivalent;
- Accommodation, education, training, employment, money, relationship and criminal related service use – self reported at baseline and 6 and 12 months post release;
- Reoffending collected using the Police National Computer Offending Data at baseline and 12 months post release.

# COST data

*Cost of the Engager Intervention*

The cost of the Engager intervention will include the cost of training and supervision divided by the number of patients per practitioner to calculate the cost per patient. The cost of providing the intervention will be based on a combination of process of care data collection and intervention practitioner care records and diaries (bottom-up costing approach), and the total costs of service provision (top-down costing). For the bottom up costing, the number and time spent on appointments and other Engager related activities will be multiplied by the average hourly cost from the most recent Unit Costs of Health and Social Care published by the Personal Social Services Research Unit (PSSRU)^2^ to calculate the average cost per patient of Engager. Additional information from the PSSRU document *Unit Costs in Criminal Justice*^3^, inflated to the same year as the most recent publication of the Unit Costs of Health and Social Care, will also be used for costing where necessary.

*Physical and mental health service resource use*

Descriptive statistics for the percentage of patients and mean number of contacts for each type of physical and mental health care resource use collected by the CSRI will be reported for patients that have completed the CSRI at baseline and 6 and/or 12 months post release. Information on data completeness will also be reported. Statistics will also be broken down by planned and unplanned health care use. Descriptive statistics will be reported (a) for patients that have completed the measures at each time point; (b) using multiple imputation for ITT analysis (see section 6 below for more details).

*Cost of health and social care service use and medication*

The cost of acute and community health care service use for the Engager versus usual care will be calculated from patient completed CSRI at baseline and 6 and 12 months post release. These will be costed for each patient using unit costs from the most recent PSSRU^2^ *Unit Costs in Criminal Justice*^3^, reference costs^4^ and published sources where needed. Costs from previous years will be inflated to the year of publication of the most recent version of the PSSRU and reference costs using the PSSRU hospital and community health services (HCHS) index^2^. Medication will be costed using the most up to date version of the British National Formulary (BNF)^5^. Mean cost per patient for the Engager versus usual care will be reported by type of service use and by planned and unplanned service use at baseline and 6 and 12 months post release and (a) for patients that have completed the measures at each time point; (b) using multiple imputation for the ITT results.

To calculate the difference in costs at 12 months between Engager and usual care, costs will be adjusted by baseline values, with study centre included as a covariate. 95% CIs will be calculated based on bootstrapped bias corrected results^6^. Only the ITT difference will be reported.

*Accommodation, education, training, employment, money, relationship and criminal related service use*

Accommodation, education, training, employment, money, relationship and criminal related service use will be calculated from patient responses baseline and 6 and 12 months post release. These will be costed for each patient using *Unit Costs in Criminal Justice*^3^ and other published sources, with all costs being inflated to the most recent year of publication of the PSSRU using the PSS Pay and Price Index^2^.

Mean cost per patient for the Engager versus usual care will be reported by type of service use at baseline and 6 and 12 months post release and (a) for patients that have completed the measures at each time point; (b) using multiple imputation for the ITT results.

The difference in costs at 12 months between Engager and usual care will be calculated with an adjustment for baseline values, with study centre included as a covariate. 95% CIs will be calculated based on bootstrapped bias corrected results^6^. Only the ITT difference will be reported.

*Reoffending*

Reoffending data will be used to calculate the patient level cost of crime in Engager compared to usual care. This will be achieved by multiplying each crime recorded in the PNC by its cost and relevant inflator index for police recorded crime as obtained from the most up to date version of *The Economic and Social Costs of Crime*^7^. All costs will be inflated to the most recent year of publication of the PPSRU using the PSS Pay and Price Index^2^. QALY losses expressed as –(QALY loss)* willingness to pay (WTP) for a QALY gained will also be calculated for each crime and for a range of values of WTP for a QALY gained.

Based on the assumption that all trial participants are successfully followed up in the Police National Computer, we will calculate the (a) mean cost per participant of crime (total crime costs divided by the number of patients randomised to each arm); (b) mean cost per participant of QALY losses (total QALYs loss times WTP for a QALY divided by the number of patients randomised to each arm); and (c) the mean cost per participant of crime and QALY losses (a+b) for Engager compared to usual care. Difference in costs between Engager and usual care and 95% CIs will be calculated based on bootstrapped^6^ bias corrected linear regression with study centre as a covariate.

# Quality of life data collection

The primary measure used to calculate QALYs will be the primary outcome, the CORE-OM, converted to the CORE-6D so that it can be used to calculate QALYs. QALYs will be calculated as the area under the curve using the CORE-6D responses at baseline and 1, 3, 6 and 12 months post release and applying by Mavranezouli et al^8^ at each time point. For the Engager versus usual care we will report the mean utility values at each time point; mean unadjusted QALYs from baseline to 12 months; and mean QALYs adjusting for baseline using regression analysis^9^. A covariate for study centre will also be included in the regression analysis.

QALYs will also be calculated reported in a similar manner using responses to the EQ-5D-5L at baseline and 3, 6 and 12 months post release and (a) the van Hout mapping algorithm to the EQ-5D-3L recommended by that National Institute of Health And Care Excellence (NICE)^10^; (b) the EQ-5D-5L value set^11^.

Years of Full Capability (YFC) (equivalent) will be calculated for Engager compared to usual care using patient level responses to the ICECAP-A at baseline and 3, 6 and 12 months post release, the tariff developed by Flynn et al^12^ and the methods for ICECAP-A and decision making set out by the University of Birmingham^13^.

95% confidence intervals for all analyses above will be calculated from bootstrapping^6^ with bias correction. Results will be reported for (a) complete cases; and (b) ITT based on section 6 below.

# Primary within-trial analysis

The primary economic evaluation will be a within-trial cost-effectiveness analysis over 12 months post release from a health and social care cost perspective.

*Incremental cost-effectiveness ratio (ICER)*

The primary result will be the mean incremental cost per QALY gained adjusting for baseline differences and with study centre as a covariate. Costs will be bootstrap adjusted costs as reported in section 3 and will include the cost of the Engager intervention in the Engager arm and the cost of health and social care services in both arms. QALYs will be bootstrap adjusted costs calculated using the CORE-6D and the methodology described in section 4. Seemlingly unrelated regression will be used to account for the correlation between costs and outcomes. The primary analysis will be based on ITT with imputation conducted as described in 6 below.

*Cost-effectiveness acceptability curve (CEAC) and Cost-effectiveness Plane*

The bootstrap results will be used to calculate the CEAC^14^: the probability that Engager is cost-effective compared to usual care for a range of values of willingness to pay for a QALY gained. A cost-effectiveness plane of the bias corrected bootstrap results will also be reported.

# MISSING DATA

The primary analysis will be ITT. For patients missing an ICER we will examine the data for predictors of missingness assuming that data are missing at random. If predictors of missingness can be identified these will be used to impute data using multiple imputation by chained equations^15^. The primary ICER, CEAC and CEP will be reported based on imputed results, seemingly unrelated regression and the methodology set out in Leurant et al^16^.

# SECONDARY WITHIN-TRIAL ANALYSES

ICERs, CEACs and CEPs will be reported for the following analyses:

i) Health and social care cost perspective using the EQ-5D-5L for the calculation of QALYS.

ii) Health and social care cost perspective using the ICECAP-A for the calculation of YFC.

iii) Health and social care, accommodation, education, training, employment, money, relationship and criminal related service use using the CORE-6D calculation of QALYS

iv) Health and social care, accommodation, education, training, employment, money, relationship and criminal related service use using the EQ-5D-5L calculation of QALYS.

v) Health and social care, accommodation, education, training, employment, money, relationship and criminal related service use using the ICECAP-A calculation of YFC.

vi) All costs including reoffending using the CORE-6D for the calculation of QALYS.

vii) All costs including reoffending using the EQ-5D-5L for the calculation of QALYS.

viii) All costs including reoffending using the ICECAP-A for the calculation of YFC.

# Cost Consequences ANALYSIS

Within the cost-consequence approach the estimated incremental health and social care costs including the cost of the Engager intervention in the treatment arm will be compared with:

- The number of people provided with the service/intervention
- Incremental differences in the number of ex-prisoners who: have resettled; are in employment; have no re-convictions; are not homeless.
- Estimated lifetime gains in Quality-Adjusted Life-Years (QALYs) – presuming the persistence of any short-term measured gains and the inclusion of estimated gains associated with social inclusion outcomes such as effective resettlement, increased employment, or reduced re-conviction rates.

Both deterministic and probabilistic sensitivity analysis will be conducted to explore uncertainty in the model assumptions and parameters, with exploration of key sources of structural uncertainty where feasible.

The analyses will be conducted according to current guidance (ISPOR) on best practice for conducting and reporting model-based economic evaluation^17^.

# Discounting

As the trial based analysis covers a 12 month duration none of the costs or quality of life outcomes will be discounting. Costs and outcomes in the cost-consequences analysis will be discounted at a rate of 3.5% in line with NICE guidance^18^.

# Sensitivity analysis

In addition to the CEAC and CEP analysis described in section 5 above, one- and two-way sensitivity analyses will be used to explore the impact of key cost assumptions on the findings. For example, some sensitivity analyses may be conducted excluding any rare but highly costly episodes of service use (for example, hospital admissions) if it seems plausible that they are unlikely to be related to the outcomes or Engager intervention and they heavily influence the magnitude of incremental costs.

Exploratory analyses will be used to investigate whether the costs associated with the Engager intervention produce a shift in the proportion of planned versus unplanned care and other service use. Exploratory analyses will also investigate whether additional costs or savings associated with having received Engager are incurred soon after release from prison, or are delayed or accrued over a longer period.

Exploratory subgroup analyses will be used to investigate whether certain groups receiving the Engager intervention account for higher or lower levels of post-intervention service use and costs; for example, those achieving stable accommodation (compared with those not); those gaining a stable occupation (compared with those not) and those obtaining ongoing care for mental health problems or substance misuse.

# References

1) Beecham JK, Knapp MRJ Costing psychiatric interventions. In: Thornicroft G, Brewin C, Wing JK (eds) *Measuring Mental Health Needs*. Gaskell: London; 1992. 200-224

2) Curtis L & Burns A. Unit Costs of Health and Social Care 2017. Personal Social Service Research Unit, University of Kent, Canterbury, 2017*.*

3) Brookes, N, Barrett B, Netten A and Knapp E. Unit Costs in Criminal Justice. Personal Social Service Research Unit, University of Kent, Canterbury, 2013

4) NHS Improvement. Reference costs 2016-2017. <https://improvement.nhs.uk/resources/reference-costs/>.

5) Joint Formulary Committee. British National Formulary (online) London: BMJ Group and Pharmaceutical Press <http://www.medicinescomplete.com>.

6) Briggs AH, Wonderling DE, Mooney CZ. Pulling cost-effectiveness analysis up by its bootstraps: a non-parametric approach to confidence interval estimation. *Health Econ* 1997;**6**:327-40.

7) Heeks M, Reed S, Tafsiri M and Prince S. The economic and social costs of crime. Second Edition. Home Office, 2018.

8) Mavranezouli, I., Brazier J.E., Rowen, D. & Barkham M. (2013) Estimating a Preference-Based Index from the Clinical Outcomes in Routine Evaluation-Outcome Measure (CORE-OM): Valuation of CORE-6D. Medical Decision Making: 33. Pp. 381-395.

9) Hunter, R. M., Baio, G., Butt, T., Morris, S., Round, J., & Freemantle, N. An Educational Review of the Statistical Issues in Analysing Utility Data for Cost-Utility Analysis. PHARMACOECONOMICS. 2015; 33 (4), 355-366.

10) National Institute for Health and Care Excellence Position statement on the use of the EQ-5D-5L valuation set for England (update November 2018) <https://www.nice.org.uk/about/what-we-do/our-programmes/nice-guidance/technology-appraisal-guidance/eq-5d-5l>

11) Devlin NJ, Shah KK, Feng Y, Mulhern B, van Hout B. Valuing health‐related quality of life: An EQ‐5D‐5L value set for England. *Health Economics*, 2018; **7-22.**

12) Flynn, TN., Huynh, E., Peters, TJ., Al-Janabi, H., Clemens, S., Moody, A. & Coast, J. (2015) Scoring the ICECAP-A capability instrument. Estimation of a UK general population tariff. Health Economics: 24. Pp. 258-269.

13) University of Birmingham. Use of ICECAP in decision making. <https://www.birmingham.ac.uk/research/activity/mds/projects/HaPS/HE/ICECAP/decision-making/index.aspx>

14) Fenwick E, Claxton K, Sculpher M. Representing Uncertainity: The role of cost-effectiveness acceptability curves. Health Econ. 2001; 10:779-87.

15) Van Buuren S, Boshuizen HC, Knook DL. Multiple imputation of missing blood pressure covariates in survival analysis. Stat Med. 1999;18(6):681-94.

16) Leurent B, Gomes M, Faria R, Morris S, Grieve R, Carpenter JR. Sensitivity Analysis for Not-at-Random Missing Data in Trial-Based Cost-Effectiveness Analysis: A Tutorial. Pharmacoeconomics. 2018;1–13. Available from: http://link.springer.com/10.1007/s40273-018-0650-5.

17) Caro JJ, Briggs AH, Siebert U, et al. Modeling good research practices - overview: A report of the ISPOR-SMDM modeling good research practices task force-1. Value Health 2012;15:796-803.

18) National Institute for Health and Care Excellence. Guide to The Methods Of Technology Appraisal. London: NICE; 2013.
