# Supplementary material for: Economic evaluation of a complex intervention (Engager) for prisoners with common mental health problems, near to and after release: a cost-utility and cost-consequences analysis
Source: Eur J Health Econ. 2021 Aug 5;23(2):193–210. doi: 10.1007/s10198-021-01360-7 (PMC8882099; doi:10.1007/s10198-021-01360-7)
Supplement: Supplementary file 2 — Supplementary material 2 (PDF 438 kb) [file 10198_2021_1360_MOESM2_ESM.pdf]

Supplementary Materials 2: Resource use descriptive statistics; number and percentage who use the resource and Mean and Standard Deviation (SD) for those who used the resource.

	**Engager**					**Usual care**				
	**n**	**n=yes**	**%**	**Mean***	**SD**	**n**	**n=yes**	**%**	**Mean***	**SD**
**Emergency mental health attendance**										
Baseline	140	18	13%	1.56	.92	140	9	6%	1.44	.88
6 months	92	14	15%	1.36	.84	140	9	6%	1.11	.33
12 months	66	10	15%	1.3	.95	58	3	5%	1.	.
**Emergency mental health admissions**				**Mean LOS**					**Mean LOS**	
Baseline	140	12	9%	4.17	4.67	140	4	3%	1.75	1.5
6 months	92	5	5%	4.2	4.55	140	4	3%	3.	3.37
12 months	66	7	11%	20.86	31.45	58	1	2%	1.	
**Planned mental health attendances**										
Baseline	140	3	2%	1.	.	140	3	2%	1.	.
**Emergency physical health attendances**										
Baseline	140	32	23%	1.28	.52	140	19	14%	1.	.
6 months	92	21	23%	1.95	2.01	90	14	16%	1.36	.74
12 months	66	24	36%	1.17	.38	58	20	34%	1.4	.82
**Emergency Physical health admissions**				**Mean LOS**					**Mean LOS**	
Baseline	140	11	8%	3.36	2.42	140	5	4%	2.	1.
6 months	92	9	10%	3.67	3.32	90	5	6%	33.6	61.93
12 months	66	6	9%	3.67	2.5	58	4	7%	6.75	9.54
**Planned physical health attendances**										
Baseline	140	10	7%	1.4	.7	140	5	4%	1.	.
6 months	92	1	1%	1.		90	2	2%	1.5	.71
12 months	66	8	12%	1.	.	58		0%		
**Planned physical health admissions**				**Mean LOS**					**Mean LOS**	
Baseline	140	2	1%	5.5	6.36	140		0%		
6 months	92	1	1%	3.		90	1	1%	35.	
12 months	66	3	5%	5.67	2.08	58		0%		
**Outpatient appointments**										
Baseline	140	12	9%	1.33	.78	140	11	8%	1.	.
6 months	92	11	12%	2.73	2.94	90	13	14%	1.69	1.25
12 months	66	9	14%	1.78	.83	58	9	16%	2.11	2.98

* Mean and Standard deviation (SD) for those that used the resource.

	Engager					Usual care				
	n	n=yes	%	Mean*	SD	n	n=yes	%	Mean*	SD
**Pre-release Service Use**										
Pre-release	115	108	94%	28.17	43.75	113	101	89%	19.07	33.5
**Community Mental Health**										
Baseline	140	84	60%	8.93	15.29	140	86	61%	7.6	12.56
6 months	92	62	67%	14.32	19.01	90	60	67%	19.45	37.91
12 months	66	45	68%	12.71	18.03	58	34	59%	16.82	29.49
**Community Physical Health**										
Baseline	140	112	80%	7.37	17.24	140	106	76%	7.5	21.77
6 months	92	73	79%	5.64	5.55	90	57	63%	6.93	13.38
12 months	66	42	64%	7.5	9.4	58	35	60%	6.89	7.05
**CJS Service Use**										
Baseline	140	59	42%	5.32	5.6	140	68	49%	2.84	3.32
6 months	92	87	95%	13.18	11.39	90	81	90%	12.31	9.32
12 months	66	47	71%	11.11	10.03	58	46	79%	11.83	12.18
**Accommodation Services**										
Baseline	140	69	49%	4.1	7.71	140	69	49%	5.87	18.87
6 months	92	49	53%	6.29	7.73	90	41	46%	5.2	6.85
12 months	66	27	41%	11.96	39.83	58	20	34%	4.85	6.18
**Education services**										
Baseline	140	99	71%	25.48	45.94	140	91	65%	23.18	30.02
6 months	92	29	32%	8.55	10.66	90	24	27%	7.04	10.39
12 months	66	18	27%	5.89	6.41	58	21	36%	6.67	9.11
**Financial advice**										
Baseline	140	22	16%	1.45	.96	140	24	17%	2.21	2.02
6 months	92	38	41%	4.29	5.1	90	34	38%	3.18	3.43
12 months	66	20	30%	5.45	6.12	58	16	28%	3.25	3.77
**Relationships**										
Baseline	140	36	26%	4.61	4.65	140	41	29%	3.2	3.37
6 months	92	9	10%	4.33	4.36	90	14	16%	4.5	5.39
12 months	66	10	15%	5.8	7.61	58	10	17%	13.2	16.07
**Other services**										

* Mean and Standard deviation (SD) for those that used the resource.

	Engager					Usual care				
	**n**	**n=yes**	**%**	**Mean***	**SD**	**n**	**n=yes**	**%**	**Mean***	**SD**
Baseline	140	117	84%	2.74	2.77	140	99	71%	3.54	5.23
6 months	92	54	59%	7.96	13.96	90	40	44%	7.38	15.67
12 months	66	27	41%	9.63	20.36	58	26	45%	10.31	25.64
**Prison**				**Mean LOS**					**Mean LOS**	
Baseline	140	13	9%	50.85	25.	140	11	8%	58.27	23.12
6 months	92	38	41%	96.34	58.4	90	27	30%	97.67	64.9
12 Months	66	28	42%	97.96	75.26	58	17	29%	84.71	59.22
**Police Contacts**										
Baseline	140	75	54%	1.81	1.38	140	65	46%	1.71	2.47
6 months	92	48	52%	1.02	.14	90	28	31%	1.04	.19
12 months	66	21	32%	1.	.	58	16	28%	1.06	.25
**Employment**				**Mean Hours Worked p/week**					**Mean Hours Worked p/week**	
Baseline	140	35	25%	36.29	16.51	140	55	39%	46.31	19.09
6 months	92	18	20%	41.08	22.78	90	24	27%	36.46	16.85
12 Months	66	14	21%	36.21	20.56	58	9	16%	35.56	23.31
**Days off work**										
Baseline	140	35	25%	4.53	11.27	140	55	39%	3.78	13.03
6 months	92	18	20%	2.7	8.74	90	24	27%	2.66	6.65
12 Months	66	14	21%	6.71	20.21	58	9	16%	1.9	3.52
**Duration of employment (weeks)**										
6 months	92	18	20%	18.39	11.22	90	24	27%	15.08	9.28
12 Months	66	14	21%	16.75	14.27	58	9	16%	26.44	16.39

SD - Standard Deviation; LOS - Length of Stay; CJS - Criminal Justice Services

* Mean and Standard deviation (SD) for those that used the resource.
