# Supplementary material for: Economic evaluation of a complex intervention (Engager) for prisoners with common mental health problems, near to and after release: a cost-utility and cost-consequences analysis
Source: Eur J Health Econ. 2021 Aug 5;23(2):193–210. doi: 10.1007/s10198-021-01360-7 (PMC8882099; doi:10.1007/s10198-021-01360-7)
Supplement: Supplementary file 3 — Supplementary material 3 (DOCX 58 kb) [file 10198_2021_1360_MOESM3_ESM.docx]

**Supplementary Material 3**

Table SM1– Complete Case mean cost per participant and adjusted difference.

|  | Engager | | | Usual Care | | |  |  |  |  |
| --- | --- | --- | --- | --- | --- | --- | --- | --- | --- | --- |
|  | n | mean | SD | n | mean | SD | Adjusted difference^a^ | 95% CI Lower | 95% CI Upper | p value |
| **Specialist Mental Health** |  |  |  |  |  |  |  |  |  |  |
| Baseline | 140 | 183 | 870 | 140 | 32 | 149 |  |  |  |  |
| 6 months | 92 | 119 | 593 | 90 | 63 | 331 |  |  |  |  |
| 12 months | 66 | 881 | 5166 | 58 | 13 | 58 |  |  |  |  |
| Total | 60 | 981 | 5412 | 54 | 112 | 424 | 854.744 | -615.426 | 2324.914 | 0.252 |
| **Physical Health Inpatient-planned** |  |  |  |  |  |  |  |  |  |  |
| Baseline | 140 | 74 | 508 | 140 | 32 | 233 |  |  |  |  |
| 6 months | 92 | 18 | 175 | 90 | 234 | 2146 |  |  |  |  |
| 12 months | 66 | 206 | 922 | 58 | 0 | 0 |  |  |  |  |
| Total | 60 | 226 | 965 | 54 | 14 | 100 | 213.168 | -50.680 | 477.017 | 0.112 |
| **Physical Health Inpatient-unplanned** |  |  |  |  |  |  |  |  |  |  |
| Baseline | 140 | 180 | 592 | 140 | 72 | 255 |  |  |  |  |
| 6 months | 92 | 260 | 823 | 90 | 226 | 1210 |  |  |  |  |
| 12 months | 66 | 257 | 920 | 58 | 306 | 1279 |  |  |  |  |
| Total | 60 | 413 | 1301 | 54 | 590 | 1940 | -236.815 | -833.296 | 359.667 | 0.433 |
| **Outpatient appointments** |  |  |  |  |  |  |  |  |  |  |
| Baseline | 140 | 23 | 102 | 140 | 11 | 47 |  |  |  |  |
| 6 months | 92 | 50 | 250 | 90 | 39 | 201 |  |  |  |  |
| 12 months | 66 | 27 | 95 | 58 | 56 | 269 |  |  |  |  |
| Total | 60 | 94 | 317 | 54 | 76 | 290 | 11.193 | -102.883 | 125.269 | 0.846 |
| **Community Health Care** |  |  |  |  |  |  |  |  |  |  |
| Baseline | 140 | 738 | 2014 | 140 | 557 | 1145 |  |  |  |  |
| Pre Release | 110 | 370 | 827 | 102 | 299 | 612 |  |  |  |  |
| 6 months | 92 | 1034 | 1487 | 90 | 920 | 1753 |  |  |  |  |
| 12 months | 66 | 940 | 1691 | 58 | 1299 | 3143 |  |  |  |  |
| Total | 46 | 2519 | 3270 | 41 | 3555 | 5030 | -776.579 | -2441.87 | 888.711 | 0.361 |
| **Medication** |  |  |  |  |  |  |  |  |  |  |
| Baseline | 140 | 37 | 73 | 140 | 57 | 188 |  |  |  |  |
| 12 months | 113 | 231 | 1994 | 111 | 58 | 215 | 153.315 | -223.313 | 529.942 | 0.423 |
| **Total health care** |  |  |  |  |  |  |  |  |  |  |
| Baseline | 140 | 2179 | 5551 | 140 | 1086 | 1979 |  |  |  |  |
| 6 months | 92 | 1727 | 2241 | 90 | 1634 | 3514 |  |  |  |  |
| 12 months | 60 | 2890 | 5888 | 56 | 1762 | 3608 |  |  |  |  |
| Total | 46 | 5174 | 7503 | 41 | 4901 | 6258 | 459.312 | -2280.11 | 3198.739 | 0.742 |
| **Criminal Justice Service Use inc. Probation** |  |  |  |  |  |  |  |  |  |  |
| Baseline | 140 | 94 | 537 | 140 | 30 | 97 |  |  |  |  |
| Pre Release | 110 | 35 | 294 | 102 | 40 | 376 |  |  |  |  |
| 6 months | 92 | 118 | 154 | 90 | 135 | 272 |  |  |  |  |
| 12 months | 66 | 95 | 169 | 58 | 195 | 780 |  |  |  |  |
| Total | 46 | 284 | 516 | 41 | 504 | 1124 | -243.008 | -644.594 | 158.578 | 0.236 |
| **Prison** |  |  |  |  |  |  |  |  |  |  |
| Baseline | 140 | 528 | 1850 | 140 | 512 | 1893 |  |  |  |  |
| 6 months | 92 | 4453 | 6771 | 90 | 3279 | 6385 |  |  |  |  |
| 12 months | 66 | 4651 | 7698 | 58 | 2778 | 5592 |  |  |  |  |
| Total | 60 | 9636 | 13073 | 54 | 5750 | 10016 | 3799.415 | -488.324 | 8087.154 | 0.082 |
| **Police** |  |  |  |  |  |  |  |  |  |  |
| Baseline | 140 | 1879 | 3171 | 140 | 1562 | 5552 |  |  |  |  |
| 6 months | 92 | 2224 | 5678 | 90 | 1113 | 4212 |  |  |  |  |
| 12 months | 66 | 2478 | 12624 | 58 | 835 | 2060 |  |  |  |  |
| Total | 60 | 5112 | 15237 | 54 | 2151 | 5561 | 3062.806 | -1200.72 | 7326.33 | 0.157 |
| **Total CJS** |  |  |  |  |  |  |  |  |  |  |
| Baseline | 140 | 2501 | 3813 | 140 | 2105 | 5859 |  |  |  |  |
| 6 months | 92 | 6795 | 9401 | 90 | 4527 | 8004 |  |  |  |  |
| 12 months | 66 | 7224 | 15450 | 58 | 3808 | 6071 |  |  |  |  |
| Total | 46 | 14260 | 16231 | 41 | 9397 | 13902 | 4854.617 | -1597.565 | 11306.799 | 0.140 |
| **Accommodation** |  |  |  |  |  |  |  |  |  |  |
| Baseline | 140 | 762 | 2442 | 140 | 735 | 2878 |  |  |  |  |
| Pre-release | 110 | 5 | 15 | 102 | 4 | 14 |  |  |  |  |
| 6 months | 92 | 2827 | 7349 | 91 | 2503 | 7634 |  |  |  |  |
| 12 months | 66 | 3086 | 9473 | 61 | 3356 | 9159 |  |  |  |  |
| Total | 55 | 5983 | 12270 | 54 | 6970 | 15861 | -1265.509 | -6804.432 | 4273.415 | 0.654 |
| **Productivity** |  |  |  |  |  |  |  |  |  |  |
| Baseline | 140 | 1780 | 3683 | 140 | 3819 | 5506 |  |  |  |  |
| 6 months | 92 | 3057 | 8404 | 90 | 2625 | 6457 |  |  |  |  |
| 12 months | 66 | 2529 | 7124 | 58 | 2640 | 8286 |  |  |  |  |
| Total | 60 | 6637 | 13110 | 54 | 4628 | 12200 | 4398.993 | 597.403 | 8200.584 | 0.023 |
| **Education** |  |  |  |  |  |  |  |  |  |  |
| Baseline | 140 | 682 | 2069 | 140 | 629 | 1727 |  |  |  |  |
| Pre-release | 110 | 514 | 1349 | 102 | 465 | 1185 |  |  |  |  |
| 6 months | 92 | 110 | 459 | 90 | 53 | 323 |  |  |  |  |
| 12 months | 66 | 91 | 375 | 58 | 24 | 89 |  |  |  |  |
| Total | 46 | 722 | 1304 | 41 | 643 | 1164 | 52.667 | -484.036 | 589.369 | 0.847 |
| **Other services** |  |  |  |  |  |  |  |  |  |  |
| Baseline | 140 | 297 | 468 | 140 | 568 | 3139 |  |  |  |  |
| Pre-release | 110 | 53 | 137 | 102 | 42 | 103 |  |  |  |  |
| 6 months | 92 | 486 | 1196 | 90 | 212 | 437 |  |  |  |  |
| 12 months | 66 | 343 | 1347 | 58 | 372 | 1143 |  |  |  |  |
| Total | 46 | 1187 | 2964 | 41 | 764 | 1433 | 356.859 | -524.969 | 1238.687 | 0.428 |
| **All costs minus productivity** |  |  |  |  |  |  |  |  |  |  |
| Baseline | 140 | 4640 | 8454 | 140 | 1304 | 9673 |  |  |  |  |
| Pre-release | 110 | 977 | 1613 | 102 | 851 | 1434 |  |  |  |  |
| 6 months | 92 | 8887 | 16998 | 90 | 6315 | 15104 |  |  |  |  |
| 12 months | 66 | 10915 | 20650 | 58 | 6741 | 15878 |  |  |  |  |
| Total | 46 | 19928 | 30925 | 41 | 18937 | 29604 | -1027.67 | -13520.59 | 11465.25 | 0.87 |

a Adjusted difference: adjusted for baseline, with centre as a covariate. Based on 5,000 bootstrap iterations.

SD – Standard Deviation; CI – Confidence interval; CJS – Criminal Justice System

Table SM2: Complete case utilities, capability and QALYs,

|  | Engager | | | Usual Care | | |  |  |  |  |
| --- | --- | --- | --- | --- | --- | --- | --- | --- | --- | --- |
|  | n | mean | SD | n | mean | SD | Adjusted difference^a^ | 95% CI  Lower | 95% CI Upper | p value |
| **CORE-6D** |  |  |  |  |  |  |  |  |  |  |
| Baseline | 140 | .75 | .168 | 140 | .713 | .181 |  |  |  |  |
| 1 month | 80 | .746 | .215 | 75 | .774 | .198 |  |  |  |  |
| 3 months | 82 | .746 | .211 | 86 | .781 | .172 |  |  |  |  |
| 6 months | 94 | .753 | .197 | 90 | .795 | .178 |  |  |  |  |
| 12 months | 68 | .764 | .22 | 60 | .738 | .236 |  |  |  |  |
| QALYs | 42 | .74 | .194 | 31 | .729 | .185 | -0.018 | -0.099 | 0.064 | 0.672 |
| **EQ-5D-5L Crosswalk** | |  |  |  |  |  |  |  |  |  |
| Baseline | 140 | .679 | .234 | 140 | .657 | .225 |  |  |  |  |
| 3 months | 82 | .687 | .266 | 86 | .685 | .274 |  |  |  |  |
| 6 months | 94 | .677 | .259 | 89 | .711 | .261 |  |  |  |  |
| 12 months | 62 | .734 | .265 | 58 | .682 | .291 |  |  |  |  |
| QALYs | 44 | .676 | .218 | 48 | .666 | .222 | -0.028 | -0.102 | 0.045 | 0.449 |
| **EQ-5D-5L Tariff** | |  |  |  |  |  |  |  |  |  |
| Baseline | 140 | .767 | .186 | 140 | .754 | .182 |  |  |  |  |
| 3 months | 82 | .766 | .243 | 86 | .766 | .226 |  |  |  |  |
| 6 months | 94 | .752 | .242 | 89 | .782 | .22 |  |  |  |  |
| 12 months | 62 | .804 | .237 | 58 | .764 | .272 |  |  |  |  |
| QALYs | 44 | .76 | .2 | 48 | .748 | .209 | -0.012 | -0.075 | 0.050 | 0.701 |
| **ICECAP-A** |  |  |  |  |  |  |  |  |  |  |
| Baseline | 140 | .613 | .221 | 139 | .613 | .226 |  |  |  |  |
| 3 months | 80 | .634 | .222 | 86 | .658 | .254 |  |  |  |  |
| 6 months | 92 | .656 | .21 | 88 | .708 | .233 |  |  |  |  |
| 12 months | 60 | .717 | .233 | 56 | .728 | .231 |  |  |  |  |
| YFC | 42 | .661 | .162 | 46 | .703 | .199 | -0.038 | -0.094 | 0.018 | 0.184 |

a Adjusted difference: adjusted for baseline, with centre as a covariate. Based on 3,000 bootstrap iterations.

SD – Standard Deviation; CI – Confidence interval; QALYs- Quality Adjusted Life Years; YFC – Years of Full Capability
