# Supplementary material for: Economic evaluation of a complex intervention (Engager) for prisoners with common mental health problems, near to and after release: a cost-utility and cost-consequences analysis
Source: Eur J Health Econ. 2021 Aug 5;23(2):193–210. doi: 10.1007/s10198-021-01360-7 (PMC8882099; doi:10.1007/s10198-021-01360-7)
Supplement: Supplementary file 4 — Supplementary material 4 (DOCX 442 kb) [file 10198_2021_1360_MOESM4_ESM.docx]

**Supplementary Material 4: Secondary and Sensitivity analyses**


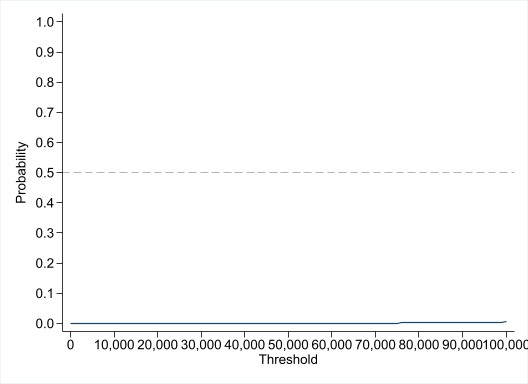


**Figure SM1:** Cost-effectiveness acceptability curve of Engager compared to usual care from a health and social care cost-perspective over 12 months with QALYs calculated using the CORE-6D.

**Secondary Analysis 1a: EQ-5D-5L, Crosswalk Tariff, health care cost perspective.**

Using the EQ-5D-5L crosswalk tariff to calculate QALYs over 12 months and from a health and social care cost perspective with MICE used for missing cost and utility data and seemingly unrelated regression to account for correlation between costs and outcomes, with adjustment for baseline and site there was a mean cost difference of £2,301 (95% of iterations between £1,289 to £3,439) and a mean QALY difference of 0.010 (95% of iterations between -0.016 to 0.036) with an incremental cost-effectiveness ratio of £237,707. The CEAC and CEP are reported in Figure SM2-SM3. There is a 0% probability that the intervention is cost-effective for a £20,000 and £30,000 threshold for a QALY gained.


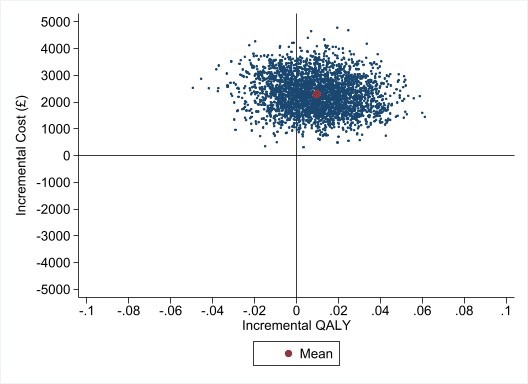


**Figure SM2:** Cost-effectiveness plane of Engager compared to usual care from a health and social care cost-perspective over 12 months with QALYs calculated using the EQ-5D-5L crosswalk tariff.

**
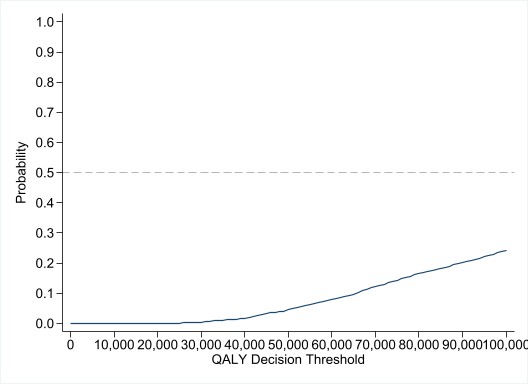
**

**Figure SM3:** Cost-effectiveness acceptability curve of Engager compared to usual care from a health and social care cost-perspective over 12 months with QALYs calculated using the EQ-5D-5L crosswalk tariff.

**Secondary Analysis 1b: EQ-5D-5L, time trade off tariff, health care cost perspective.**

Using the EQ-5D-5L time trade off tariff to calculate QALYs over 12 months and from a health and social care cost perspective with MICE used for missing cost and utility data and seemingly unrelated regression to account for correlation between costs and outcomes, with adjustment for baseline and site there was a mean cost difference of £2,301 (95% of iterations between £1,293 to £3,444) and a mean QALY difference of 0.001 (95% of iterations between -0.021 to 0.024) with an incremental cost-effectiveness ratio of £2,301,000. The CEAC and CEP are reported in Figure SM4-SM5. There is a 0.2% and 1% probability that the intervention is cost-effective for a £20,000 and £30,000 threshold for a QALY gained, respectively.
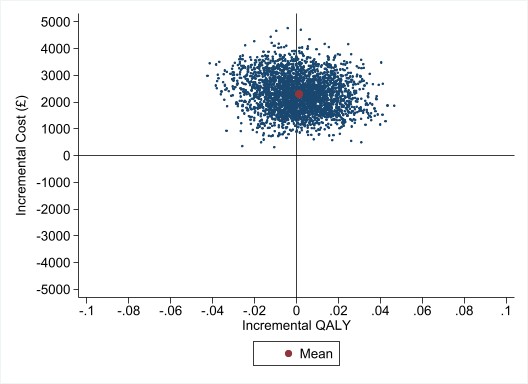


**Figure SM4:** Cost-effectiveness plane of Engager compared to usual care from a health and social care cost-perspective over 12 months with QALYs calculated using the EQ-5D-5L utility tariff.


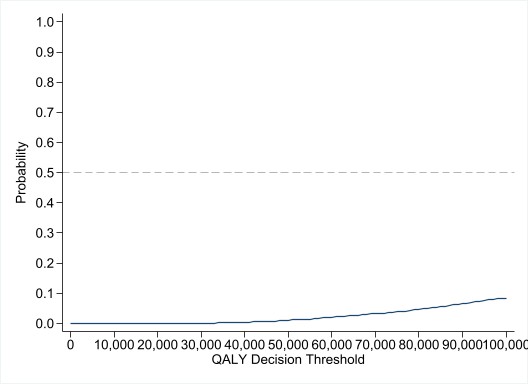


**Figure SM5:** Cost-effectiveness acceptability curve of Engager compared to usual care from a health and social care cost-perspective over 12 months with QALYs calculated using the EQ-5D-5L utility tariff.

**Secondary Analysis 2: ICECAP-A and tariff, health care cost perspective.**

Using the ICECAP-A and related tariff to calculate years of full capability (YFC) over 12 months and from a health and social care cost perspective with MICE used for missing cost and capability and seemingly unrelated regression to account for correlation between costs and outcomes, with adjustment for baseline and site there was a mean cost difference of £2,184 (95% of iterations between £1,173 to £3,354) and a mean YFC difference of -0.030 (95% of iterations between -0.061 to 0.003); Engager is dominated by usual care. The CEAC and CEP are reported in Figure SM6-SM7. There is a 0% probability that the intervention is cost-effective for a £20,000 and £30,000 threshold for a QALY gained.

**
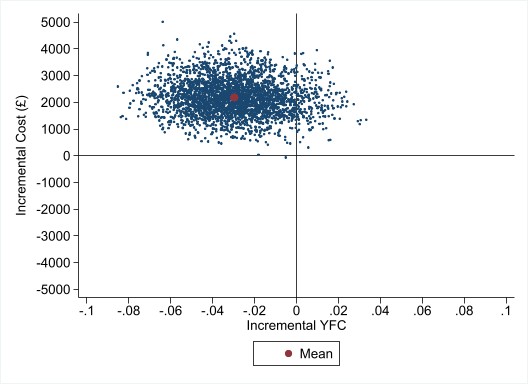
**

**Figure SM6:** Cost-effectiveness plane of Engager compared to usual care from a health and social care cost-perspective over 12 months with YFC calculated using the ICECAP-A tariff.


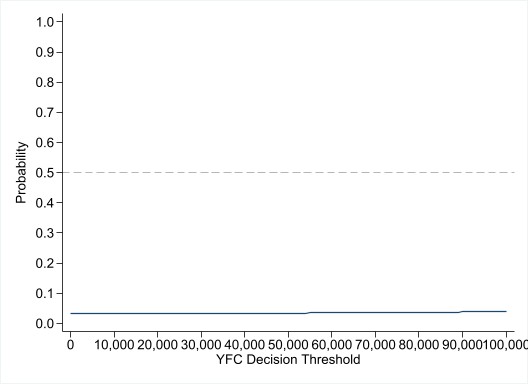


**Figure SM7:** Cost-effectiveness acceptability curve of Engager compared to usual care from a health and social care cost-perspective over 12 months with YFC calculated using the ICECAP-A tariff.

**Secondary Analysis 3: CORE-6D and tariff, wider cost perspective.**

Using the CORE-6D tariff to calculate QALYs over 12 months and from a health and social care cost perspective with MICE used for missing cost and utility data and seemingly unrelated regression to account for correlation between costs and outcomes, with adjustment for baseline and site there was a mean cost difference of £6,428 (95% of iterations between -£1,308 to £13,894) and a mean QALY difference of -0.015 (95% of iterations between -0.046 to 0.016); Engager is dominated by usual care. The CEAC and CEP are reported in Figure SM8-SM9. There is an 8% probability that the intervention is cost-effective for a £20,000 and £30,000 threshold for a QALY gained.


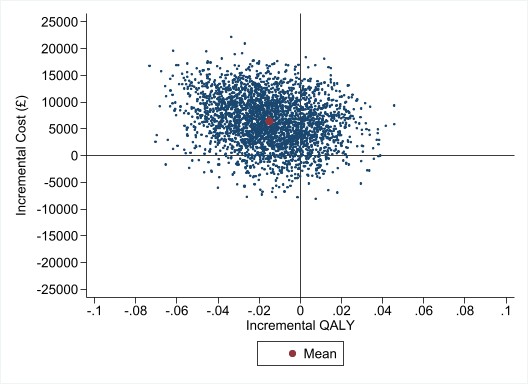


**Figure SM8:** Cost-effectiveness plane of Engager compared to usual care from a wider cost-perspective over 12 months with QALYs calculated using the CORE-6D tariff.


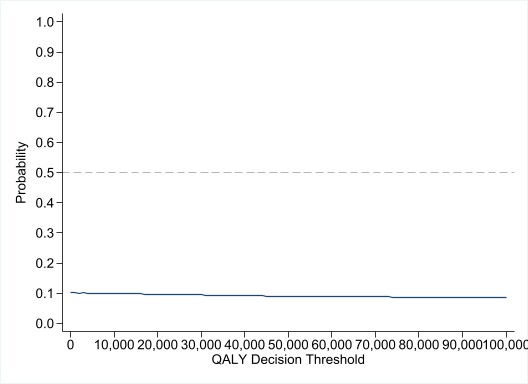


**Figure SM9:** Cost-effectiveness plane of Engager compared to usual care from a wider cost-perspective over 12 months with QALYs calculated using the CORE-6D tariff.

**Secondary Analysis 4a: EQ-5D-5L, Crosswalk Tariff, health care cost perspective.**

Using the EQ-5D-5L crosswalk tariff to calculate QALYs over 12 months and from a wider cost perspective with MICE used for missing cost and utility data and seemingly unrelated regression to account for correlation between costs and outcomes, with adjustment for baseline and site there was a mean cost difference of £4,775 (95% of iterations between -£1,370 to £10,231) and a mean QALY difference of 0.003 (95% of iterations between -0.025 to 0.035) with an incremental cost-effectiveness ratio of £1,591,667. The CEAC and CEP are reported in Figure SM10-SM11. There is an 11% probability that the intervention is cost-effective for a £20,000 and £30,000 threshold for a QALY gained.


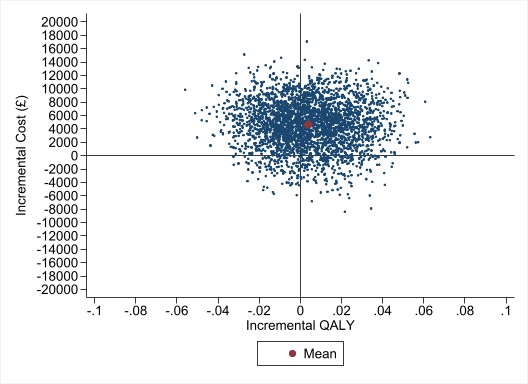


**Figure SM10:** Cost-effectiveness plane of Engager compared to usual care from a wider cost-perspective over 12 months with QALYs calculated using the EQ-5D-5L crosswalk tariff.


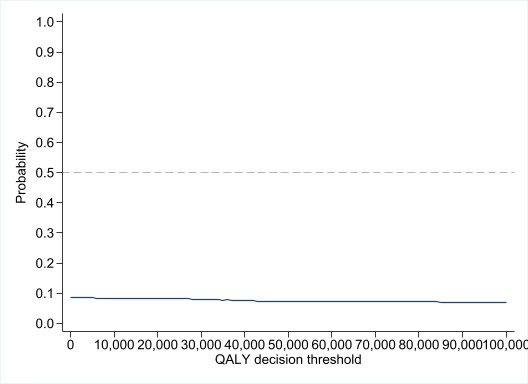


**Figure SM11:** Cost-effectiveness acceptability curve of Engager compared to usual care from a wider cost-perspective over 12 months with QALYs calculated using the EQ-5D-5L crosswalk tariff.

**Secondary Analysis 4b: EQ-5D-5L, time trade off tariff, health care cost perspective.**

Using the EQ-5D-5L time trade off tariff to calculate QALYs over 12 months and from a health and social care cost perspective with MICE used for missing cost and utility data and seemingly unrelated regression to account for correlation between costs and outcomes, with adjustment for baseline and site there was a mean cost difference of £4,758 (95% of iterations between -£1,400 to £10,208) and a mean QALY difference of -0.007 (95% of iterations between -0.034 to 0.020); Engager is dominated by usual care. The CEAC and CEP are reported in Figure SM12-SM13. There is a 10% probability that the intervention is cost-effective for a £20,000 and £30,000 threshold for a QALY gained.

**
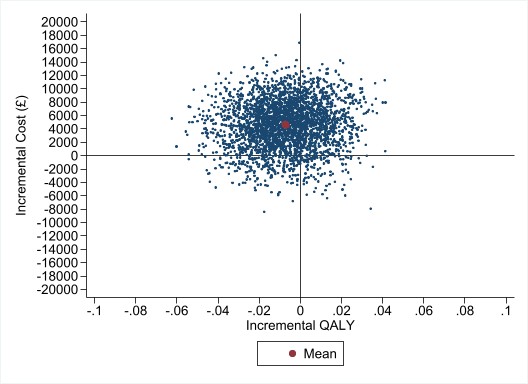
**

**Figure SM12:** Cost-effectiveness plane of Engager compared to usual care from a wider cost-perspective over 12 months with QALYs calculated using the EQ-5D-5L utility tariff.


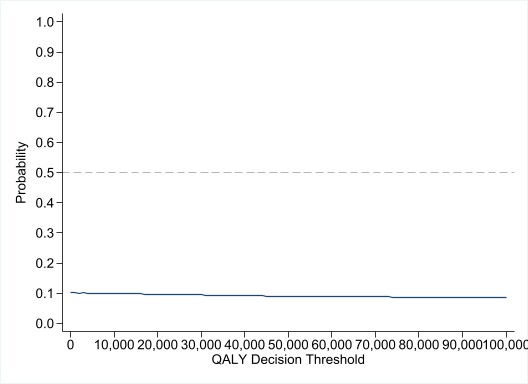


**Figure SM13:** Cost-effectiveness acceptability curve of Engager compared to usual care from a wider cost-perspective over 12 months with QALYs calculated using the EQ-5D-5L utility tariff.

**Secondary Analysis 2: ICECAP-A and tariff, health care cost perspective.**

Using the ICECAP-A and related tariff to calculate years of full capability (YFC) over 12 months and from a health and social care cost perspective with MICE used for missing cost and capability and seemingly unrelated regression to account for correlation between costs and outcomes, with adjustment for baseline and site there was a mean cost difference of £5,391 (95% of iterations between -£1,239 to £11,084) and a mean YFC difference of -0.030 (95% of iterations between -0.063 to 0.003); Engager is dominated by usual care. The CEAC and CEP are reported in Figure SM14-SM15. There is a 7% probability that the intervention is cost-effective for a £20,000 and £30,000 threshold for a QALY gained.

**
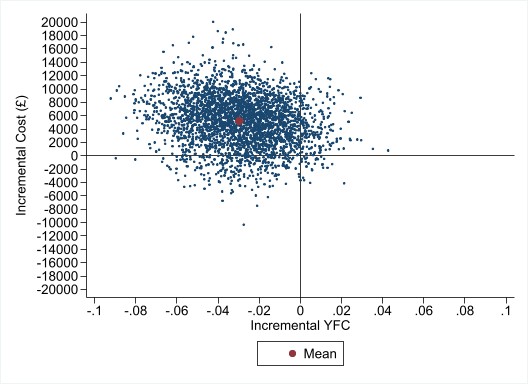
**

**Figure SM14:** Cost-effectiveness plane of Engager compared to usual care from a wider cost-perspective over 12 months with YFC calculated using the ICECAP-A tariff.


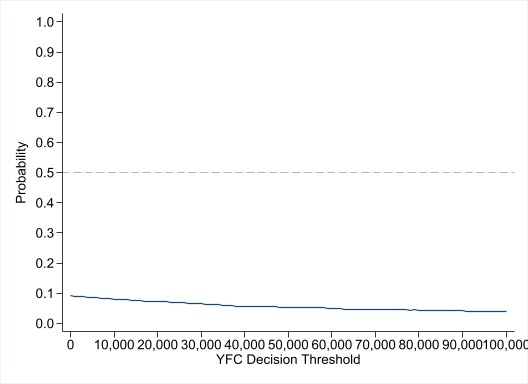


**Figure SM15:** Cost-effectiveness acceptability curve of Engager compared to usual care from a wider cost-perspective over 12 months with YFC calculated using the ICECAP-A tariff.

**Sensitivity analysis 1:** If the cost of the Engager intervention is calculated using top-down costs, and costs an additional £3784 per participant randomised to Engager, the total additional cost of the Engager intervention from a health care perspective is £5272 (95% CI £3747 to £6799) per participant, adjusting for baseline costs and centre and using MICE to account for missing data.

**Sensitivity analysis 2:** If meta-supervision is removed from training and supervision costs, the total cost of training and supervision is £54,563, or £392 per participant. The total additional cost of the Engager intervention from a health care cost-perspective is £2,201 (95% CI £787 to £3615) per participant, adjusting for baseline costs and centre and using MICE to account for missing data.

**Sensitivity analysis 3:** If practitioner supervision is reduced from weekly to fortnightly, the total cost of training and supervision is £42,759, or £305 per participant. The total additional cost of the Engager intervention from a health care cost-perspective is £2,114 (95% CI £700 to £3528) per participant, adjusting for baseline costs and centre and using MICE to account for missing data.

**Sensitivity analysis 4:** If pre-release health care costs, ones that the intervention potentially had no ability to influence, are removed from the total health care costs, the total additional cost of the Engager intervention from a health care cost-perspective is £2,971 (95% CI £1605 to £4337) per participant, adjusting for baseline costs and centre and using MICE to account for missing data.

**Sensitivity analysis 5:** When the duration from randomisation until prison release is included as covariate in the analysis the mean difference in QALYs calculated using the CORE-6D is -0.017 (95% CI -0.053 to 0.019) per participant, with a total additional mean cost of the Engager intervention from a health care cost-perspective of £2,119 (95% CI £686 to £3552) per participant, adjusting for baseline and centre and using MICE to account for missing data.
